# Ethylene Receptors, CTRs and EIN2 Target Protein Identification and Quantification Through Parallel Reaction Monitoring During Tomato Fruit Ripening

**DOI:** 10.3389/fpls.2018.01626

**Published:** 2018-11-08

**Authors:** Clara I. Mata, Bertrand Fabre, Harriet T. Parsons, Maarten L. A. T. M. Hertog, Geert Van Raemdonck, Geert Baggerman, Bram Van de Poel, Kathryn S. Lilley, Bart M. Nicolaï

**Affiliations:** ^1^Postharvest Group, Division of Mechatronics, Biostatistics and Sensors, Department of Biosystems, KU Leuven, Leuven, Belgium; ^2^Cambridge Centre for Proteomics, Cambridge Systems Biology Centre, University of Cambridge, Cambridge, United Kingdom; ^3^Centre for Proteomics and Mass Spectrometry, University of Antwerp, Antwerp, Belgium; ^4^Flemish Institute for Technological Research (VITO), Mol, Belgium; ^5^Molecular Plant Hormone Physiology, Division of Crop Biotechnics, Department of Biosystems, KU Leuven, Leuven, Belgium

**Keywords:** ethylene signal transduction, ethylene receptors, targeted proteomics, parallel reaction monitoring, ripening, tomato

## Abstract

Ethylene, the plant ripening hormone of climacteric fruit, is perceived by ethylene receptors which is the first step in the complex ethylene signal transduction pathway. Much progress has been made in elucidating the mechanism of this pathway, but there is still a lot to be done in the proteomic quantification of the main proteins involved, particularly during fruit ripening. This work focuses on the mass spectrometry based identification and quantification of the ethylene receptors (ETRs) and the downstream components of the pathway, CTR-like proteins (CTRs) and ETHYLENE INSENSITIVE 2 (EIN2). We used tomato as a model fruit to study changes in protein abundance involved in the ethylene signal transduction during fruit ripening. In order to detect and quantify these low abundant proteins located in the membrane of the endoplasmic reticulum, we developed a workflow comprising sample fractionation and MS analysis using parallel reaction monitoring. This work shows the feasibility of the identification and absolute quantification of all seven ethylene receptors, three out of four CTRs and EIN2 in four ripening stages of tomato. In parallel, gene expression was analyzed through real-time qPCR. Correlation between transcriptomic and proteomic profiles during ripening was only observed for three of the studied proteins, suggesting that the other signaling proteins are likely post-transcriptionally regulated. Based on our quantification results we were able to show that the protein levels of SlETR3 and SlETR4 increased during ripening, probably to control ethylene sensitivity. The other receptors and CTRs showed either stable levels that could sustain, or decreasing levels that could promote fruit ripening.

## Introduction

Worldwide, tomato is the second most important vegetable crop in terms of production (Food and Agriculture Organization of United Nations, 2016). It is widely used as a model organism to study fleshy fruit development and climacteric fruit ripening (Giovannoni, 2004; Osorio et al., 2011). The ripening of tomato, and of climacteric fruit in general, is regulated by the plant hormone ethylene, which also regulates numerous aspects of plant growth and development including responses to biotic and abiotic stress (Van de Poel et al., 2015; Wen, 2015). Climacteric fruit is characterized by a burst in respiration which coincides with a burst in ethylene production at the onset of ripening, decreasing both afterward when the fruit becomes ripe (Lelievre et al., 1997).

Post-harvest control of ethylene is of great importance to assure proper storage conditions and to control fruit quality. Thus, a good understanding of ethylene perception by the fruit is essential to eventually improve post-harvest practices. The ethylene signal transduction pathway starts with the perception of ethylene by a family of receptors spanning the membrane of the endoplasmic reticulum (Chen et al., 2002; Zhong et al., 2008). In tomato there are seven ethylene receptors (ETRs), with the seventh only recently been validated by phylogenetic analysis (Wilkinson et al., 1995; Lashbrook et al., 1998; Tieman and Klee, 1999; Klee and Tieman, 2002; Liu et al., 2015). The receptors are homologous to bacterial two-component histidine kinases, formed of a sensory histidine kinase and a response regulator domain (Chang et al., 1993). Depending on their histidine kinase activity, the receptors have been classified into two subfamilies. Three ethylene receptors (SlETR1-SlETR3) are classified into subfamily I containing a well-conserved histidine kinase domain, and four receptors (SlETR4-SlETR7) into subfamily II, missing some of the residues to act as histidine kinases (Klee, 2002; Liu et al., 2015). Mutant analyses have shown that the receptors are negative regulators of the ethylene response, meaning that in the presence of ethylene the receptors are inactivated, which leads to the induction of ethylene signaling (Hua and Meyerowitz, 1998; Tieman et al., 2000). The ethylene receptors interact with the downstream CTR-like protein kinases (Zhong et al., 2008). Four of these tomato CTR-like proteins are homologous to the Raf-like kinase CONSTITUTIVE TRIPLE RESPONSE1 of *Arabidopsis*, which is also a negative regulator of the ethylene response (Kieber et al., 1993; Adams-Phillips et al., 2004; Zhong et al., 2008). ETRs maintain the conformation of CTR1, which in this state is able to phosphorylate and inhibit ETHYLENE INSENSITIVE 2 (EIN2), another endoplasmic reticulum spanning protein (Ju et al., 2012; Qiao et al., 2012; Wen et al., 2012). The generally accepted model is that ethylene binding to the receptors reduces their phosphorylation levels, which results in receptor degradation through the proteasome (Chen et al., 2007; Kevany et al., 2007; Kamiyoshihara et al., 2012). As a consequence, CTR1 is inactivated and EIN2 ceases to be phosphorylated, which results in the cleavage and translocation of the EIN2 C-terminal part to the nucleus (Ju et al., 2012; Qiao et al., 2012; Wen et al., 2012). In the nucleus the C-terminal part of EIN2 stabilizes EIN3 and EIN3-like proteins (EILs), preventing them from proteasomal degradation mediated by the F-box proteins ETHYLENE BINDING FACTOR 1 (EBF) and EBF2 (Guo and Ecker, 2003; An et al., 2010). Alternatively, the EIN2-C terminal end can also control ethylene sensitivity via a non-nuclear mechanism, through the translational repression of EBF1 and EBF2 synthesis (Li et al., 2015; Merchante et al., 2015). The nuclear transcription factors EIN3 and EILs promote the expression of ethylene response factor (ERF) family genes which are downstream regulators of the ethylene responses (Fujimoto et al., 2000; Tieman et al., 2001; Tournier et al., 2003; Liu et al., 2016).

Several studies have analyzed gene expression of the ethylene receptors during tomato fruit ripening, showing, in general, an increase in expression at the onset of ripening for *SlETR3*, *SlETR4*, and *SlETR6* (Kevany et al., 2007; Rugkong et al., 2011; Osorio et al., 2012; Liu et al., 2015). Recently, Mata et al. (2018) showed peaks in expression at the onset of ripening for the receptors *SlETR2*-*SlETR6* and *SlCTR1* and *SlCTR2.* Previous transcriptional analysis of *CTRs* in tomato revealed that only *SlCTR1* was ethylene induced during ripening, while the *SlEIN2* expression levels, which are not so well-documented, did not change during ripening (Zegzouti et al., 1999; Leclercq et al., 2002; Adams-Phillips et al., 2004; Lin et al., 2008; Liu et al., 2015). Recent studies have shown that the transcribed mRNA and translated protein are not necessarily directly correlated, as changes in gene expression are frequently not reflected at the protein level (Ghazalpour et al., 2011; Vogel and Marcotte, 2012; Peng et al., 2015). This might be due to factors such as different half-lives, post-transcriptional modifications or protein degradation, amongst others. Therefore, transcript analyses need to be supplemented by protein quantification to fully understand the underlying regulation. To date, only three ethylene receptor proteins have been quantified in tomato pericarp through western blot analyses. Two studies showed high protein levels for SlETR3 (also called Never Ripe), SlETR4 and SlETR6 in immature fruit, which significantly decreased during the onset of ripening (Kevany et al., 2007, 2008), while a third study showed increasing protein levels for SlETR3 and SlETR4 during ripening (Kamiyoshihara et al., 2012).

Western blotting, a semi-quantitative technique, is a common method to quantify proteins through the binding of specific antibodies (Towbin et al., 1979). However, the assay relies on the specificity of the antibodies which can be limited by cross-reactivity and unspecific binding to other proteins, leading to the production of an imprecise identification and quantification (Mann, 2008; Liebler and Zimmerman, 2013). Furthermore, the quality of the antibodies cannot always be easily verified. Nowadays, liquid chromatography mass spectrometry (LC–MS) provides an improved alternative to western blotting in terms of protein identification and quantification as it measures multiple signals (multiple peptides per protein, multiple fragment ions per peptide, and multiple measurements of each signal) as opposed to the intensity of a single band. Moreover, mass spectrometry has the power of multiplexing, that is, to simultaneously measure multiple proteins in a single run at high-throughput.

A few LC–MS discovery studies in *Arabidopsis* have found, among the total proteins identified, some AtETRs, AtCTRs, and AtEIN2 (Maor et al., 2007; Baerenfaller et al., 2008, 2011; Marondedze et al., 2014). Chen et al. (2011) and Qiao et al. (2012) used mass spectrometry to specifically study the cleavage site of AtEIN2 and its phosphorylation status. Recently, two studies have identified some of the ethylene signaling elements in tomato through mass spectrometry (Mata et al., 2017; Szymanski et al., 2017). Both studies used an untargeted data dependent acquisition (DDA) approach. Shotgun proteomics attempts to identify and quantify as many proteins as possible, but is inherently biased toward the most abundant peptides (Gilmore and Washburn, 2010). To focus on the identification and quantification of low abundant proteins, targeted proteomics techniques such as selected reaction monitoring (SRM) and parallel reaction monitoring (PRM) have been developed (Lange et al., 2008; Peterson et al., 2012). These techniques have become the gold standard in targeted proteomics (Gillet et al., 2012; Aebersold and Picotti, 2013). Unlike in shotgun proteomics, in SRM and PRM acquisition modes, peptides of interest must be defined in advance. The first mass analyzer selects a narrow mass window around the m/z of the ions of interest, thereby discarding other ions and thus increasing the signal to noise ratio (Liebler and Zimmerman, 2013). In PRM mode all transitions are acquired and measured in the second mass analyzer, while in SRM mode an extra selection of the transitions to be measured in the MS2 is applied (Gallien et al., 2012; Peterson et al., 2012). Moreover, synthetic peptides with an amino acid sequence identical to the targeted peptides are used for a first identification screening, while spiking of the samples with a known concentration of isotopically labeled peptides can deliver absolute peptide quantification (Kirkpatrick et al., 2005).

The objective of the present work was to develop a targeted LC–MS based method to identify and quantify ethylene receptors, CTRs and EIN2 proteins of the ethylene signal transduction pathway in tomato pericarp, to study their dynamics during fruit ripening and eventually their regulation at the gene expression level. Up to date, this work has not been done due to the difficulty of the identification of such very low abundant proteins (Mata et al., 2017). Our previous results from an extensive LC–MS shotgun approach (Mata et al., 2017) were taken as a starting point. In this targeted assay, a specific microsomal membrane protein extraction followed by fractionation of the protein samples through SDS-PAGE was used to reduce the complexity of the tomato pericarp samples. After protein digestion, the peptides were analyzed on the LC–MS in PRM mode to be able to counteract the low abundance problem. Subsequently, the proteins were absolutely quantified in tomato fruit of four different ripening stages using heavy labeled peptides. To complement the proteomics data, gene expression of the targeted proteins was investigated using real-time qPCR. This enabled a comparison of protein abundance and gene expression levels for the targeted proteins of the ethylene signal transduction pathway during tomato fruit ripening.

## Materials and Methods

### Plant Material

Tomato plants (*Solanum lycopersicum* L. cv. Bonaparte) were grown in a greenhouse at the Research Station for Vegetable Production of Sint-Katelijne-Waver (Belgium). Plants were hydroponically cultivated on rockwool under natural light. Twelve biological replicates from each maturity stage (mature green, breaker, orange, and red) were harvested (April 2016) after visual inspection. Mature green corresponded to fully developed tomatoes that had not started the ripening process yet; breaker, to tomatoes in which ripening was initiated and the first degreening was visible; orange, to the ones in which no green color was visible anymore and red tomatoes, to the ones which matched the final red-ripe stage. Pericarp tissue of 24 tomatoes (six biological replicates for each ripening stage) were directly homogenized and processed for protein extraction. The pericarp tissues of the other 24 samples (six biological replicates for each ripening stage) were flash frozen in liquid nitrogen, crushed with a grindomixer (Retsch, Haan, Germany) and stored at -80°C for gene expression analysis.

### Protein Extraction

The protein extraction method was adapted from Kamiyoshihara et al. (2012). The pericarp tissue of each sample was homogenized at 4°C using a high speed disperser (IKA Labortechnik, Staufen, Germany) in 2 volumes of homogenization buffer (100 mM Tris-HCl [pH 8.2], 300 mM NaCl, 20 mM EDTA, 20% [v/v] glycerol, 5 mM dithiothreitol [DTT] with complete EDTA-free protease inhibitor cocktail [Roche, Basel, Switzerland]), and centrifuged at 5,000 *g* for 15 min at 4°C. The supernatants were filtered over Miracloth (Merc Millipore, Darmstadt, Germany), and centrifuged at 100,000 *g* for 1 h at 4°C. The pellets were re-suspended in homogenization buffer containing 10% SDS, 10 mM Tris pH 7.5, and the samples were boiled at 95°C for 5 min. Protein concentrations of solubilized pellets were determined with a DC protein assay kit (Bio-Rad, Hercules, CA, United States) using bovine serum albumin as standard.

### Reduction Alkylation, SDS-PAGE Fractionation and In-Gel Digestion

Hundred μg of protein per sample were denaturated and reduced by addition of Laemmli buffer for 5 min at 95°C and then alkylated by addition of 60 mM iodoactetamide for 30 min at RT in the dark. The samples were loaded on an SDS-PAGE gel (4% stacking and 12% resolving) and were migrated until the smallest protein band of the pre-stained protein standard (New England BioLab, Ipswich, MA, United States) reached the end of the gel. Proteins were stained overnight with colloidal blue Coomassie staining. For each gel lane, one band fraction containing the proteins ranging from 163 to 52 kDa was excised from the gel and cut into small pieces. Gel pieces were de-stained in 25 mM ammonium bicarbonate/50% acetonitrile (ACN) at 37°C, then incubated in ACN for 15 min. Gel pieces were dried in a speed-vac until the ACN was completely evaporated. Gel pieces were incubated overnight in 500 ng of trypsin in 50 mM ammonium bicarbonate at 37°C. Next, 200 μL 10% formic acid (FA) and 200 μL 100% ACN were added to the gel pieces and incubated during 15 min at 37°C. The supernatant was retained, and gel pieces were re-incubated with 200 μL 100% ACN and 200 μL 10% FA. Supernatants were pooled and dried in a speed-vac. Finally, the pellets were re-suspended in 2% ACN and 0.1% FA and the peptide concentration determined with a Pierce Quantitative Colorimetric Peptide Assay (Thermo Scientific, Waltham, MA, United States).

### Design of the Targeted Proteomics Experiment

Parallel reaction monitoring assays were developed using Skyline version 4.1 (University of Washington, United States, MacLean et al., 2010). *In silico* tryptic digestions of protein sequences obtained from UniProt (Bateman et al., 2015) were performed. Target peptides were selected using the following criteria: peptide mass between 7 and 25 amino acids, no missed cleavages, absence of methionines, cysteines, and histidines and RP KP (prolines after the arginines and lysines). Modifications were set to carbamidomethylation of cysteines, oxidation of methionines and N-terminal acetylation, tolerating three possible modifications per peptide and one neutral loss. Uniqueness of the targets was verified using the tomato proteome (downloaded from UniProt on December 2015, 40,069 sequences, Bateman et al., 2015). The following settings were used to select the transitions: precursor charges 2 and 3, ion charges 1 and 2, ion types y, b, and p (precursor), 3 product ions from m/z to precursor, ion match tolerance 0.5 Da, pick 10 product ions, isotope peaks included COUNT, precursor mass analyzer Orbitrap, peaks 3, resolving power 60,000 at m/z 400, acquisition method targeted, product mass analyzer Orbitrap, use only scans within 5 min of MS–MS IDs.

### Non-labeled and Labeled Synthetic Peptides

Unlabeled synthetic peptides (SpiketidesTM) for assay development were purchased from JPT Innovative Peptide Solutions (Berlin, Germany, Schnatbaum et al., 2011). A list with all the unlabeled peptides tested can be found in Supplementary Table 1. The labeled peptides (SpikeTides_TQL) for the combined identification and quantification, purchased from the same company, were heavy-isotope labeled on the C-terminal lysine or arginine and absolutely quantified using a proprietary Quanti-Tag. Table 1 presents the list of peptides monitored for the quantification and their corresponding labeled peptides. The proteotypic labeled peptides were pooled and digested with trypsin to be released from the tag.

**Table 1:** List of the proteins identified and quantified, their peptides and their corresponding labeled peptides monitored in PRM analysis, the precursor’s m/z and charge, the product ions used for the quantification, the average retention time (RT) of their extracted ion peaks and the amount of labeled peptide in fmol used to spike into the samples for the quantification of the endogenous peptide.

Protein	Peptide sequence	Precursor m/z (charge state)	Product ions for PRM	RT	Amount of labeled peptide used for quantification (fmol)
ETR1	ISPNSPVAR ISPNSPVA[Heavy R]	470.7642 ( + 2) 475.7683 ( + 2)	y7^+^, y6^+^, y5^+^, y3^+^, y7^++^,	13.25	10
	EGNVSISAFVAK EGNVSISAFVA [Heavy K]	611.3273 ( + 2) 615.3344 ( + 2)	Y8^+^, y7^+^, y6^+^	23.30	50
ETR2	YIPGEVVAVR YIPGEVVAV[Heavy R]	551.8164 ( + 2) 556.8205 ( + 2)	y8^+^, y7^+^, y6^+^, y5^+^, y4^+^, y8^++^	20.47	5
ETR3	YIPPEVVAVR YIPPEVVAV[Heavy R]	571.8320 ( + 2) 576.8362 ( + 2)	y8^+^, y7^+^, y6^+^, y5^+^, y4^+^, y8^++^, y7^++^, b2^+^	21.69	10
	VPLLHLSNFTNDWAELSTR VPLLHLSNFTNDWAELST[Heavy R]	738.3832 ( + 3) 741.7193 ( + 3)	y8^+^, y7^+^, y6^+^, y5^+^, y4^+^, y3^+^, b12^++^	34.25	100
	LIQTLLNVAGNAVK LIQTLLNVAGNAV[Heavy K]	727.4405 ( + 2) 731.4476 ( + 2)	y12^+^, y10^+^, y9^+^, y8^+^, y7^+^, y4, y3^+^, b4^+^, b5^+^	30.31	400
ETR4	DSSFNSAYNLPIPR DSSFNSAYNLPIP[Heavy R]	790.8888 ( + 2) 795.8929 ( + 2)	y9^+^, y8^+^, y7^+^, y4^+^	29.25	15
	SDPDVIQVK SDPDVIQV[Heavy K]	500.7691 ( + 2) 504.7762 ( + 2)	y7^+^, y6^+^, y5^+^, y7^++^	16.15	15
	VLPESVSR VLPESVS[Heavy R]	443.7533 ( + 2) 448.7574 ( + 2)	y6^+^, y5^+^, y4^+^, y6^++^	14.61	10
ETR5	SLSINDPDVLEITK SLSINDPDVLEIT[Heavy K]	772.4143 ( + 2) 776.4214 ( + 2)	y9^+^, y8^+^, y7^+^	28.93	50
ETR6	FWLNQEVEIVR FWLNQEVEIV[Heavy R]	716.8828 ( + 2) 721.8869 ( + 2)	y8^+^, y7^+^	31	25
	GVEVLLADYDDSNR GVEVLLADYDDSN[Heavy R]	783.3757 ( + 2) 788.3799 ( + 2)	y9^+^, y8^+^, y7^+^	27.9	100
ETR7	SLPIDDPDVLEITK SLPIDDPDVLEIT[Heavy K]	777.9167 ( + 2) 781.9238 ( + 2)	y9^+^, y8^+^, y12^++^	30.51	15
	GLQVLLADDDDVNR GLQVLLADDDDVN[Heavy R]	771.8916 ( + 2) 776.8957 ( + 2)	y9^+^, y8^+^, y7^+^, b8^+^	25.98	100
CTR1	IPSIESLR IPSIESL[Heavy R]	457.7689 ( + 2) 462.7731 ( + 2)	y7^+^, y6^+^, y5^+^, y4^+^, y7^++^	21.5	15
	LNPPQVIAAVGFNR LNPPQVIAAVGFN[Heavy R]	748.4226 ( + 2) 753.4268 ( + 2)	y10^+^, y9^+^, y8^+^, y12^++^, y11^++^	29.76	15
CTR2	YAPNEVPR YAPNEVP[Heavy R]	473.2431 ( + 2) 478.2472 ( + 2)	y6^+^, y5^+^, y4^+^, y6^++^	14.35	10
	LVIPAYVDQLNSR LVIPAYVDQLNS[Heavy R]	744.4145 ( + 2) 749.4186 ( + 2)	y10^+^, y9^+^, y8^+^, y7^+^, y10^++^, b3^+^	28.38	10
CTR3	ASASAASAETLSHR ASASAASAETLSH[Heavy R]	679.8366 ( + 2) 684.8407 ( + 2)	y8^+^, y7^+^, y6^+^	12.78	5
EIN2	GVSENAQSFISDGPGSYK GVSENAQSFISDGPGSY[Heavy K]	921.9289 ( + 2) 925.9360 ( + 2)	y11^+^, y10^+^, y9^+^, y5^+^	23.66	50
	VESSAYIPSGSAR VESSAYIPSGSA[Heavy R]	662.3306 ( + 2) 667.3347 ( + 2)	y9^+^, y8^+^, y7^+^, y6^+^	16.31	5


### LC–MS and Parallel Reaction Monitoring (PRM) Acquisition

Samples (1 μg) were analyzed in PRM acquisition mode on a Q Exactive Plus mass-spectrometer (Thermo Scientific, Waltham, MA, United States), using a 75 μm × 2 cm, C18, 3 μm, 100 Ȧ trapping column (Acclaim PepMap, Thermo Scientific) and an Easy nLC 1000 system (Thermo Scientific). Peptides were separated with a 50 μm × 15 cm, nanoViper, C18, 2 μm, 100 Ȧ column (Acclaim PepMap) retrofitted to a NanoSpray Flex source with a flow rate of 300 nL/min (buffer A: HPLC grade H_2_O, 0.1% FA, buffer B: 100% ACN, 0.1% FA). Samples were run using a 60 min gradient from 5% up to 35% solvent B. Analytes were transferred to the gaseous phase with positive ion electrospray ionization at 2.0 kV. Precursors were targeted with a 2 m/z selection window around the m/z of interest. Precursors were fragmented in high-energy collisional dissociation mode with normalized collision energy of 28. A single MS1 scan was performed at a mass resolution of 17,500, an automatic gain control (AGC) target of 10^6^ ions and a maximum C-trap fill time of 200 ms. Subsequently, 10 PRM scans were performed at a resolution of 70,000, an AGC target of 10^5^ ions and a maximum injection time of 200 ms. Initial screening for targets transitions was unscheduled but retention-time scheduling of PRM (sPRM) was adopted for peptide quantification, allowing analysis of 42 peptides in a single acquisition.

### Provisional Peptide Identification

For the first screening and provisional identification of the endogenous peptides in the samples, a PRM analysis of a pooled sample of the unlabeled peptides was performed, followed by PRM analyses of endogenous peptides from tomato samples. The individual raw-files were imported into Skyline, and precursor and product ion chromatograms were extracted. MS–MS spectra were analyzed in Skyline with manual validation comparing the extracted ion chromatogram (XIC) of the unlabeled peptides and the endogenous peptides of the tomato sample. Peptide identification was based on retention time, the presence of the main transition ions and a low mass error (less than 5 ppm). Labeled synthetic versions were ordered for candidate peptides with the most consistently detectable transitions.

### Preparation of the Labeled Synthetic Peptides Mix

The labeled synthetic peptides were spiked into endogenous peptides digests (six aliquots of 1 μg) from tomato samples at the following concentrations: 0, 1, 5, 10, 100, and 200 fmol. The aliquots were measured by LC–MS in PRM mode using retention time scheduling. Based on a comparison of the XIC of the labeled and endogenous peptides, final concentrations of labeled peptides were chosen for absolute quantitation experiments such that signal intensity was similar to that of endogenous peptides.

This experiment was also used to evaluate the linearity of the dilution curves for the individual peptides. The ratio of sum of the area-under-the-curve (AUC) of the transitions (Table 1) of the heavy labeled peptide to the sum of the AUC of the transitions of the endogenous peptide contained in the tomato peptide pool was calculated to correct for run to run variation of the different LC–MS analysis of the spiking concentrations. The dilutieon curves are provided as (Supplementary Figure 1).

### Peptide Identification and Quantification

After spiking the samples with labeled peptides, two sets of precursor ions were detected upon PRM analysis: heavy-isotope labeled (mass difference + 8 if containing a lysine or + 10 if containing an arginine) and non-labeled (from digested endogenous protein). The XIC from each individual peptide was manually checked in Skyline to ensure the correct identification of the peptide across biological replicates. Furthermore, the mProph algorithm was used to calculate the FDR (q-value) of the targeted peptide identifications trained with the second best peak option. The information extracted from this analysis is provided in Supplementary Table 2. It was found that 83.7% of the transitions groups were identified with q-values < 0.01 (FDR of 1%). About 13.9% had a q-value between 0.01 and 0.05, some of which were eliminated from the analysis and 2.4% displayed q-values higher than 0.05, which were directly removed. For the quantification, the ratio of sum of the AUC of the transitions of the endogenous peptide to the sum of the AUC of the transitions of the heavy labeled peptide was used to calculate the absolute concentration of the peptide in the sample, also known as single point calibration quantification (Gallien et al., 2013). Supplementary Figure 2 displays the absolute quantification of the individual peptides of the target proteins.

### RNA Extraction and cDNA Synthesis

Total RNA was extracted from tomato fruit pericarp samples. Ground tissue samples (500 mg) were homogenized in 800 μL of extraction buffer containing cetyltrimethylammonium bromide, as described previously (Gasic et al., 2004). The mixture was incubated vigorously shaking at 65°C for 10 min. Chloroform (800 μL) was added and mixed by inversion, and the mixture was centrifuged at 21,000 *g* for 10 min at room temperature. The supernatant was transferred to a gDNA eliminator spin column (Plant RNeasy Extraction Kit, Qiagen, Hilden, Germany) and centrifuged at 8,000 *g* for 2 min at room temperature. Half a volume of ethanol was added to the effluent, then the mixture was loaded and washed through the RNeasy mini column (Plant RNeasy Extraction Kit) and finally the RNA was eluted with RNAse free water. The amount of total RNA extracted was measured by spectrophotometry using the NanoDrop 2000 (Thermo Scientific, Waltham, MA, United States) and its purity determined by the 260/280 or 260/230 nm ratio. RNA integrity was checked on an ethidium bromide stained 1% agarose gel. One microgram of purified RNA was reverse transcribed into cDNA using the QuantiTect Reverse Transcription Kit (Qiagen) in a total volume of 20 μL following the manufacturer’s protocol.

### Gene Expression Analysis by Reverse Transcription-qPCR

Gene expression studies were performed following Minimum Information for publication of Quantitative Real-Time PCR Experiments (MIQE) guidelines (Bustin et al., 2009). Real-time qPCR was carried out with SYBR^®^ Green PCR Master Mix (Thermo Scientific, Waltham, MA, United States) on a Rotor Gene Q (Qiagen GmbH, Hilden, Germany). The selected primers, designed with the Primer3 web tool^1^, are listed in Supplementary Table 3. All RT-qPCR reactions contained 1 μL of cDNA template (50 mg/L), 7.5 μL of Absolute QRT-PCR SYBR Green Mix (Thermo Scientific), and 1 μL of 0.375 μM primer pairs, in a final volume of 15 μL. The cycling conditions were as follows: denaturation step at 95°C for 15 min, followed by 40 cycles of denaturation at 95°C for 20 s, annealing at 63°C for 20 s, and extension at 72°C for 20 s. Primer pair specificity was performed for every run using a melting curve analysis ranging from 55 to 95°C, with temperature increasing in steps of 0.5°C/s. Furthermore, a standard dilution curve, based on cDNA pooled from all samples, was included in every run to calculate the efficiency of the amplification. The relative quantification of expression levels was performed using a modified Ct method as previously described (Mellidou et al., 2012). All RT-qPCR expression data were normalized against the average expression of three reference genes: Actin, Elongation factor1, and Glyceraldehyde-3-phosphate dehydrogenase. Results presented are the mean ± standard error (SE) of six independent biological replicates.

### Statistical Analyses

Given an individual protein was represented by up to three different peptides, protein data were analyzed using the mixed models procedure. In this approach ‘ripening stage’ was considered a fixed categorical factor while ‘peptide’ was treated as a categorical random factor introducing a repeated structure ‘sample’ to account for the fact that the various peptides were covariates measured on the same fruit samples. In the case of a single peptide per protein the classical one-way ANOVA was applied. In both cases, Tukey’s honestly significant difference (HSD) test (*p* < 0.05) was used to compare between ripening stages. Statistical differences in gene expression between ripening stages were analyzed with the one-way ANOVA procedure and Tukey’s HSD test (*p* < 0.05). All analyses were performed using JMP 12 statistical software (SAS Institute, Cary, NC, United States).

Correlation between protein and gene expression levels was calculated and can be visualized in Supplementary Figure 3. Given protein and gene expression levels were measured on different biological replicates their structural correlation is not known. To approximate this relationship, 1500 random data sets were generated with the same distribution properties (average and standard error of the mean) based on which the correlation coefficients were calculated. Using a Fisher transformation, the 95% confidence interval was calculated and from that, the significance of the correlation coefficient was determined. The protein, gene expression data and their standard errors were normalized for visualization.

## Results

### Identification of the Proteins Through PRM

**FIGURE 1:**
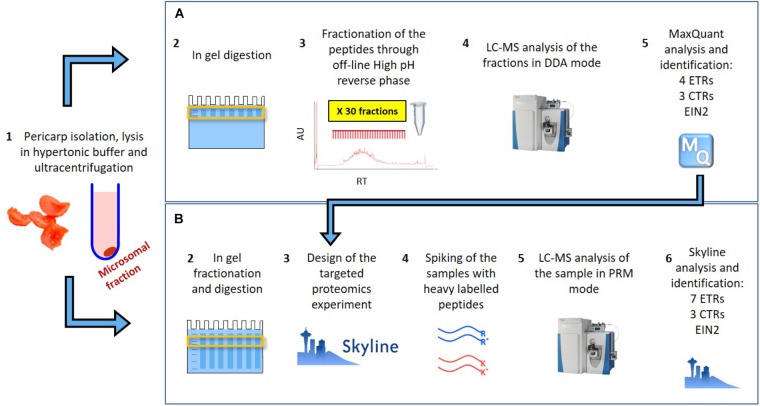
Schematic representation of **(A)** the extensive fractionation analyzed though shotgun approach which allowed to identify 8588 proteins and between them 4 ETR, 3 CTR and EIN2 in tomato pericarp (Mata et al., 2017) and **(B)** the targeted proteomics approach followed in this work which allowed the identification and quantification of the 7 ETR, 3 CTRs and EIN2.

In Mata et al. (2017) we provided the identification of 8588 tomato pericarp proteins, including four ethylene receptors (SlETR1, SlETR3, SlETR4, and ETR7), three CTRs (SlCTR1-SlCTR3) and SlEIN2. The approach taken, shown schematically in Figure 1A, consisted of the extraction of the pericarp proteins from a red tomato through a microsomal membrane isolation protocol, followed by in-gel digestion and fractionation of the subsequent peptides through off-line high pH reverse phase. The 60 sub-fractions obtained through the fractionation were analyzed on a Q Exactive and a Triple-TOF 6600 mass spectrometers in shotgun mode. This was the starting point of our current research as: (i) it allowed us to prove the identification of some of our proteins of interest through LC–MS and (ii) it helped to prioritize the peptides to follow in targeted mode.

**FIGURE 2:**
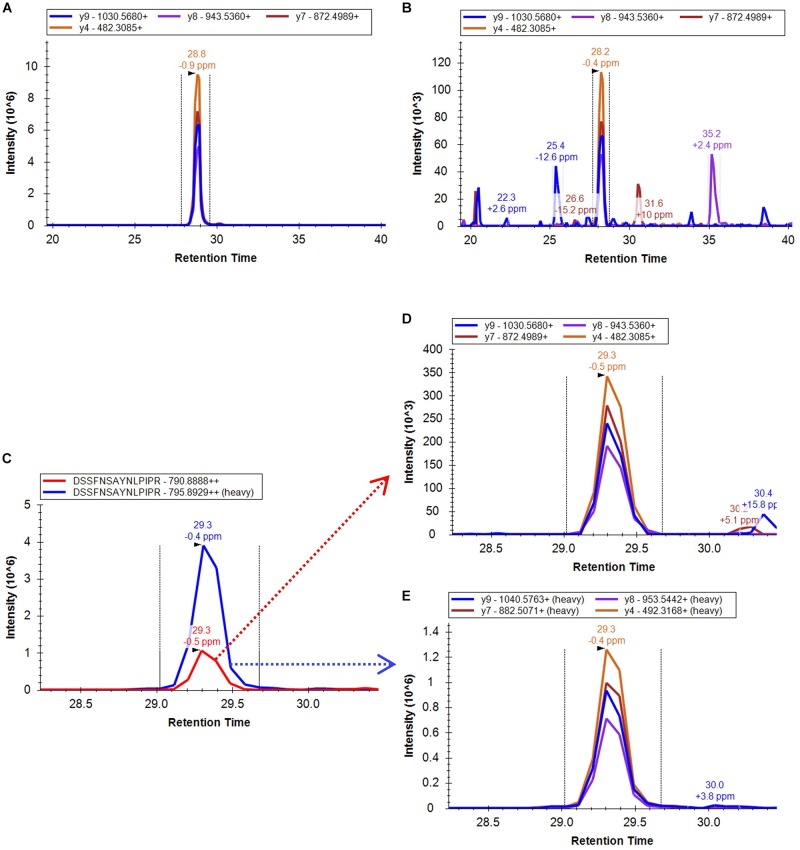
Extracted ion chromatogram of the PRM four most intense fragment ions identified from **(A)** the synthetic non-labeled peptide DSSFNSAYNLPIPR, **(B)** an endogenous peptide sample derived from a mature green tomato. **(C)** XIC of the combined fragment ions of the endogenous peptide (red peak) DSSFNSAYNLPIPR of the protein SlETR4 in a mature green tomato peptide sample and the combined fragment ions of its heavy labeled peptide (blue peak) spiked in the sample. **(D)** XIC of the four most intense fragment ions used for quantification of the endogenous peptide and **(E)** the equivalent fragments for the heavy labeled peptide. All data were analyzed by the Skyline software.

The approach taken in the current work is shown schematically in Figure 1B. After *in silico* digestion of the target proteins (7 SlETRs, 4 SlCTRs and SlEIN2) a list of unique peptides was established. Those unique peptides that also followed the criteria for being identifiable in MS1, were combined with a selection of unique peptides identified during the previous shotgun approach (Mata et al., 2017), resulting in a list of 88 unique peptides for the 12 proteins targeted (Supplementary Table 1). An unscheduled PRM analysis was conducted on unlabeled, synthetic versions of these 88 peptides. By comparing retention times, fragment ions, and mass errors of their MS2 spectra with those of native peptides derived from different ripening stages of tomato, we identified promising candidate peptides for all seven ethylene receptors, three CTRs (1–3) and EIN2 (Supplementary Table 1). This approach is exemplified in Figures 2A,B where similarities in transitions and retention times, with low mass errors, were observed between a synthetic and endogenous SlETR4 peptide.

To confirm the identification and to be able to quantify the endogenous peptides, heavy labeled C-terminal lysine or arginine peptides of 21 of the peptides candidates were ordered afterward and were spiked in tomato samples from four different ripening stages, from mature green to red. The PRM analysis of these samples proved the legitimate identification of all the 21 endogenous peptides and, therefore, of the 11 ethylene signaling proteins. The PRM.raw data and Skyline results files are available via ProteomeXchange in PeptideAtlas/PASSEL repository (PASS01249) and the output of the mProphet analysis can be found in Supplementary Table 2. An example of the XIC of the fragment ions of one of the identified peptides of the protein SlETR4 and its corresponding labeled peptide is shown in Figures 2C–E. This figure shows that retention time, fragment ions and the intensity order of the fragment ions are the same for endogenous and labeled peptide confirming its identification.

The location of the 21 peptides, used for the quantification, in the specific protein sequences can be checked in Supplementary Table 4. As it can be appreciated, the quantified peptides came from different protein domains, as in the case of SlETR1 in which one of the peptides derived from the predicted GAF domain and the other from the kinase domain. Qiao et al. (2012) revealed for *Arabidopsis* the amino acid residue where the proteolytic cleavage of the C-terminal domain of EIN2 is produced after ethylene binds to the receptor-CTR complex. We performed a Clustal alignment with UniProt between the EIN2 protein of *Arabidopsi*s and tomato and both proteins only have 48% sequence similarity (Supplementary Table 5). There is no information about the proteolytic residue of the SlEIN2, but based on the alignment, the first tomato peptide identified in this study may contain the proteolysis residue. The second tomato peptide identified likely belongs to the C-terminal end of SlEIN2.

### Absolute Quantification of the Protein Levels

**FIGURE 3:**
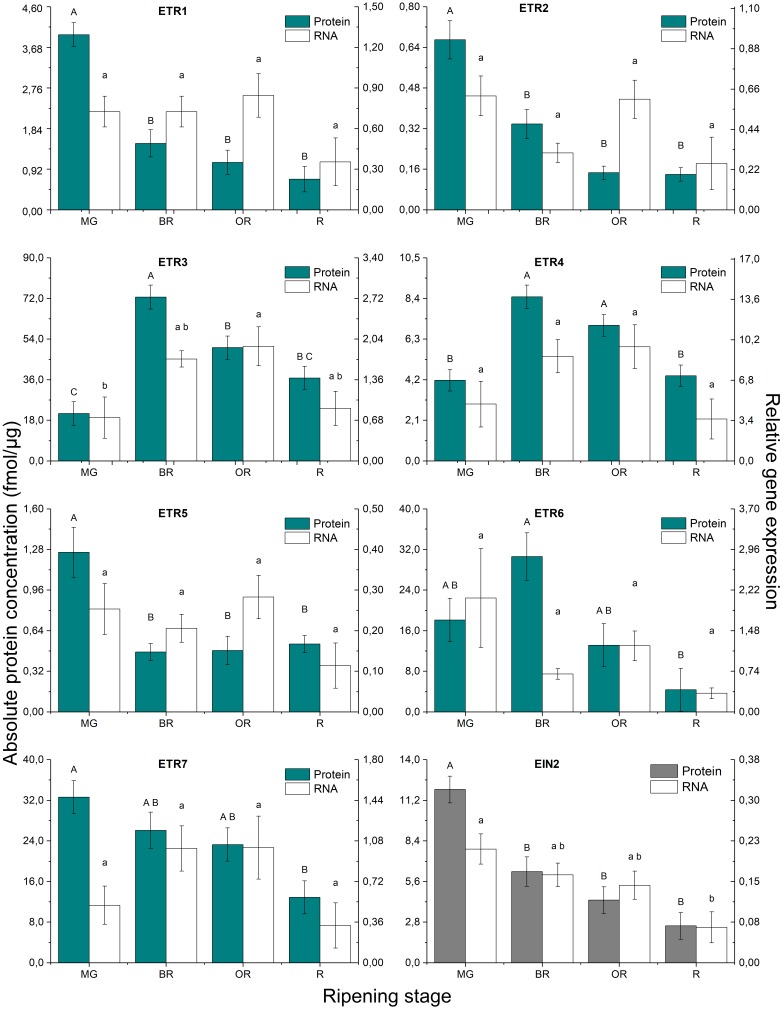
Absolute protein quantification (fmol of target protein/μg of total membrane proteins) and relative gene expression of ETR1-ETR7, and EIN2 during tomato fruit ripening. MG, mature green; BR, breaker; OR, orange; R, red tomatoes. Error bars represent the standard error of the mean based on six biological replicates. Difference uppercase letters indicate significant differences between the absolute protein concentration levels of the four tomato ripening stages determined by Tukey’s HSD test (*p* < 0.05). Different lowercase letters indicate significant differences between the relative gene expression levels of the four tomato ripening stages determined by Tukey’s HSD test (*p* < 0.05).

**FIGURE 4:**
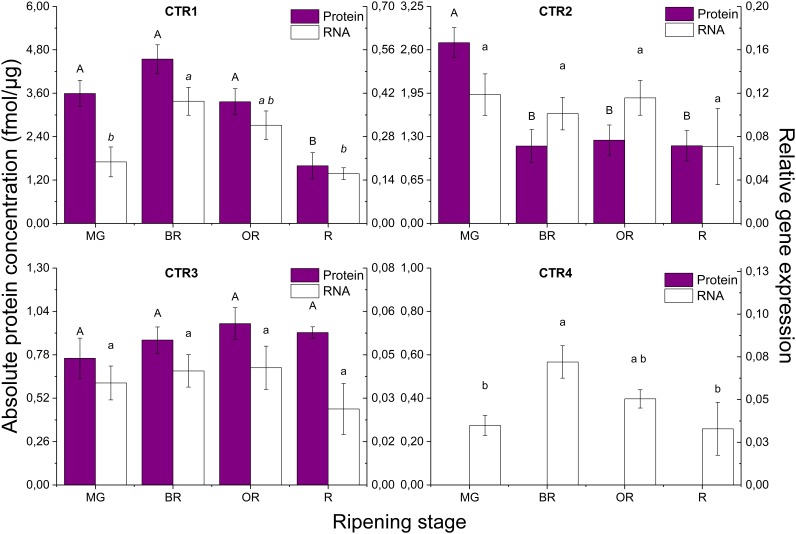
Absolute protein quantification (fmol of target protein/μg of total membrane proteins) and relative gene expression of CTR1–CTR4 during tomato fruit ripening. MG, mature green; BR, breaker; OR, orange; R, red tomatoes. Error bars represent the standard error of the mean based on six biological replicates. Difference uppercase letters indicate significant differences between the absolute protein concentration levels of the four tomato ripening stages determined by Tukey’s HSD test (*p* < 0.05). Different lowercase letters indicate significant differences between the relative gene expression levels of the four tomato ripening stages determined by Tukey’s HSD test (*p* < 0.05).

The representation of the absolute quantification of the individual peptides of the target proteins, in fmol of target protein/μg of total membrane proteins, is shown in Supplementary Figure 2. Most proteins were identified with two peptides (SlETR1, SlETR6, SlETR7, SlCTR1, SlCTR2, and SlEIN2), while some proteins with one (SlETR2, SlETR5, and SlCTR3) or three (SlETR3 and SlETR4) peptides. It can be observed that for some of the proteins identified with more than one peptide, the absolute concentration levels of their peptides are variable, highlighting the limit of absolute quantification using spiked peptides. For these proteins identified with multiple peptides, the absolute peptide quantifications were combined in a final protein quantification representation through the use of mixed models. Figures 3, 4 shows the graphical representation of the absolute protein quantification of the 11 proteins identified, for the four ripening stages of tomato, in combination with their gene expression levels measured in the same ripening stages. SlCTR4 protein levels could not be quantified, probably because of the low abundancy of this protein, so only its gene expression levels are shown (Figure 4).

Figure 3 demonstrates that the most abundant ethylene receptor proteins are SlETR3, SlETR6 and SlETR7, followed by SlETR4, SlETR1, SlETR5 and finally SlETR2. SlCTR1 is the most abundant SlCTR protein, followed by SlCTR2 and SlCTR3 (Figure 4). SlETR3 and SlETR4 are the only proteins whose abundance profiles seemed to follow a climacteric protein pattern, both increasing significantly at the onset of ripening followed by a subsequent decrease toward the red ripening stage. The receptors SlETR1, SlETR2 and SlETR5, SlCTR2 and SlEIN2 proteins are most abundant during the mature green stage, decreasing significantly at the start of ripening and maintaining low levels during the breaker, orange and red stages. On the other hand, the protein abundance of SlETR6, SlETR7, and SlCTR1 only decrease during the red ripening stage, so at the onset of ripening no significant changes are observed. SlCTR3 abundance is maintained constant throughout fruit ripening.

### Analysis of the Transcripts Levels

Figure 3 demonstrates that *SlETR4* shows the highest expression of all the *SIETRs*, followed by *SlETR3* and *SlETR6*, and then *SlETR7*, *SlETR1*, and *SlETR2*. The expression level of *SlETR5* is the lowest. Within the *SlCTRs*, *SlCTR1* and *SlCTR2* are more expressed compared to *SlCTR3* and *SlCTR4* (Figure 4). None of the *SlETRs* show significant changes in gene expression between different ripening stages, except for *SlETR3* of which transcript levels are higher in the orange ripening stage compared to the mature green fruit. Both *SlCTR1* and *SlCTR4* expression levels show a climacteric expression pattern, while *SlCTR2* and *SlCTR3* do not significantly change during the four ripening stages. The mRNA levels of *SlEIN2* are significantly higher in mature green fruit as compared to red fruit.

When comparing the correlation between gene expression and protein levels (Supplementary Figure 3) a significant correlation is found only for SlETR3, SlCTR1, and SlEIN2.

## Discussion

### Benefits and Limitations of the Ethylene Signaling Protein Quantification

Szymanski et al. (2017) performed a proteomics discovery experiment similar to the one of Mata et al. (2017) as outlined in Figure 1A, identifying SlETR3, SlETR4, SlCTR2 and SlEIN2, and showed that SlETR3 has a climacteric profile during ripening. However, such methods are not ideal for the quantification of low abundant proteins in a large number of samples, because the production and MS analysis becomes very costly due to the fractionation required. Furthermore, some of the peptides used for the identification of the proteins appeared in more than one sub-fraction which might generate quantification and reproducibility issues. However, such preliminary discovery work provided a solid starting point on which the current targeted proteomics workflow was based (Figure 1B). The current workflow provides a simplified protein fractionation step through SDS-PAGE, without the need of producing extra sub-fractions, and provides a targeted search of the proteins on the LC–MS which, thanks to the increased sensitivity and signal to noise ratio, allows the identification and quantification of low abundant proteins of interest (Gallien et al., 2012). Furthermore, it is a relatively easy and reproducible technique.

The introduction of isotopically labeled peptides provided a strong identity confidence and allowed an absolute quantification of the endogenous peptides in the sample. However, spiking of the samples can only be done just before the LC–MS analysis, and is therefore not accounting for any technical variance nor protein losses during earlier steps. As a result, the estimated absolute protein levels can still be prone to errors. For some proteins, the endogenous peptides resulted in considerably different concentrations (Supplementary Figure 2). We hypothesize that this could be due to (i) different trypsin digestion efficiency in different parts of the protein, (ii) incomplete re-solubilization of the labeled peptides during their initial preparation, and/or (iii) partial adsorption of the labeled peptides onto vials. The tryptic digestion efficiency problem would produce an underestimation of some of the endogenous peptides due to their incomplete digestion, while the incomplete re-solubilization or adsorption of the labeled peptides would cause an overestimation, as the calculated spiking concentrations would be smaller in reality. It would, therefore, be interesting to also test QCAT proteins, which is a concatenation of standard tryptic peptides encoded by an artificial gene, and PSAQ which are isotope-labeled full length proteins with the same amino acid composition as the endogenous proteins (Beynon et al., 2005; Brun et al., 2007). These proteins can be incorporated earlier during sample processing and should display biochemical properties more similar to the endogenous proteins (Brun et al., 2007).

### Ethylene Receptor Abundance Is Linked to Fruit Ripening of Tomato

Our quantitative analyses demonstrated that SlETR3, SlETR4, SlETR6, and SlETR7 were the most abundant receptors during tomato fruit ripening. Our gene expression results also showed that these receptors were the most expressed. These results are in accordance with the high expression levels for *SlETR3* and *SlETR4* observed in other studies (Kevany et al., 2007; Yan et al., 2013; Liu et al., 2015; Mata et al., 2018). It is thus plausible that these receptors are the most important and thus play an important role in regulating ethylene sensitivity during climacteric fruit ripening of tomato. Both protein abundance and gene expression data showed that SlCTR1 was the most abundant member of the SlCTR family during fruit ripening. Our gene expression data for S1CTR1 are similar to data from Adams-Phillips et al. (2004) and Liu et al. (2015). The high expression and protein abundance data for SlCTR1 might indicate that SlCTR1 is the main fruit ripening specific SlCTR member. Previous work demonstrated that transgenic antisense tomato lines with a reduced expression of *SlETR3*, *SlETR4*, and *SlETR6* showed an increased ethylene sensitivity and an accelerated ripening phenotype (Tieman et al., 2000; Kevany et al., 2007). Fu et al. (2005) also demonstrated that silencing *SlCTR1* expression using virus-induced gene silencing, promoted fruit ripening in green tomatoes. Because the receptors and SlCTRs act as negative regulators of ethylene signaling (Kieber et al., 1993; Hua and Meyerowitz, 1998; Tieman et al., 2000; Lin et al., 2008), a higher abundance of these proteins would lead to a reduced ethylene sensitivity.

Receptor phosphorylation has been also linked to ethylene sensitivity, as Kamiyoshihara et al. (2012) showed that both SlETR3 and SlETR4 are differentially phosphorylated during fruit ripening and by an ethylene, 1-MCP or 2,5-norbornadiene treatment, likely influencing receptor stability or activity. So, it seems that ethylene receptor turnover, but also receptor activity, is most likely regulated by specific post-translational modifications and by the hormone itself.

### Climacteric Protein Levels of SlETR3 and SlETR4 Control Fruit Ripening

Kevany et al. (2007) showed that an ethylene treatment of tomato resulted in a rapid decline in receptor protein abundance of SlETR3, SlETR4 and SlETR6, likely caused by protein degradation through the proteasome-dependent pathway. They also quantified receptor abundance during ripening, using western blot, and hypothesized that the decreasing protein levels during ripening were caused by receptor degradation (Kevany et al., 2007). Our mass spectrometry quantification analysis reported results more similar to the ones of Kamiyoshihara et al. (2012), which showed by western blot that SlETR3 and SlETR4 receptor abundance increased during tomato fruit ripening. Specifically, in our results SlETR3 and SlETR4 showed a peak in the protein levels, suggesting that the concentration of these proteins follows the climacteric ethylene production levels observed during ripening. This bring us to the hypothesis that receptor degradation of SlETR3 and SlETR4 after ethylene binding, cannot counteract the high synthesis rate of new receptors during the onset of ripening. Therefore, as the receptors are negative regulators of the ethylene response, both the climacteric increase in the protein levels of SlETR3 and SlETR4 and their high abundance suggest that these receptors might control and reduce ethylene sensitivity at the onset of fruit ripening and as a consequence, control the timing and rate of fruit ripening. The increase in receptor abundance during ripening may allow the fruit to bind more ethylene which is autocatalytically produced and so control ethylene sensitivity and its downstream responses. On the other hand, the drop of SlETR3 and SlETR4 receptor abundance at the end of ripening, when tomatoes have turned red, might be related to the decline in ethylene production levels after the climacteric peak. When less free ethylene is produced, fewer receptors are necessary to control ethylene sensitivity and control ripening. During this post-climacteric ripening stage, it is possible that receptor degradation is higher than *de novo* synthesis.

The positive feedback that ethylene exerts on receptor abundance during ripening is likely caused by an increase in receptor gene expression. Our results showed that the mRNA levels of *SlETR3* increased during ripening, while the mRNA levels of *SlETR4* followed a climacteric trend but did not show significant differences during ripening. However, it seems odd to find an increase in the SlETR4 protein levels during the onset of ripening without any increase in the mRNA levels (Figure 3). When studying the correlation between gene expression and protein abundance levels during ripening, only SlETR3 was significantly correlated (Supplementary Figure 3). The expression of both *SlETR3* and *SlETR4* have been studied the most during tomato fruit ripening, confirming an increase in expression during fruit ripening for both genes (Kevany et al., 2007; Osorio et al., 2012; Yan et al., 2013; Liu et al., 2015; Mata et al., 2018). Assuming a short change in gene expression can induce a longer lasting response at the protein level, our interpretation is that the current four ripening stages were too coarse to identify such short lasting significant changes at the transcript levels for SlETR4. Adding intermediate ripening stages would have helped to provide a more accurate picture of this regulation, like in the case of Mata et al. (2018).

### Steady State Protein Levels Sustain Fruit Ripening

Protein levels of the receptors SlETR6 and SlETR7 and SlCTR1 and SlCTR3 stayed constant during ripening, only showing a perceivable decrease when the fruit reached its red ripe stage, except for SlCTR3. SlETR6 protein abundance seemed to increase in breaker fruit compared to mature green, but this change was not significant. Furthermore, the gene expression levels of both receptors (SlETR6 and SlETR7) and *SlCTR3* displayed no significant changes during ripening. The correlation between gene expression and protein abundance levels was not significant either, indicating that the protein turnover is possibly driven by post-translational modifications including protein degradation, instead of by gene expression directly. A possible explanation for the trend observed for these receptors and SlCTR3 could be that constant protein levels were sustained as a mechanism to control ethylene sensitivity in a more gentle way than through receptors 3 and 4, thus they would sustain the ripening process. The final low protein levels in the red stage would again be the consequence of the end of ripening, where no extra action would be needed to control the process.

In the case of *SlCTR1* the increase in the expression levels is not reflected at the protein level. However, a significant correlation between both kind of data was found during ripening, indicating that the protein abundance was directly controlled by gene expression. We hypothesize that in this specific case, the high transcription was counteracted by a fast rate of protein degradation of the newly formed protein after the binding of ethylene to the receptor-CTR complex. This could be the reason why no peak in protein levels was observed. Given SlCTR1 is the most abundant CTR and because of its specific behavior, it might be the strongest regulator of the tomato CTRs. Likewise the transcript levels of *SlCTR4* behaved, but its low abundancy did not allow its identification in spite of using the highly sensitivity targeted acquisition proteomics method PRM.

### Decreasing Protein Levels Enable the Onset of Fruit Ripening

It is remarkable that SlETR1, SlETR2 and SlETR5 and SlCTR2 protein levels rapidly declined as soon as ripening started in the breaker stage. However, no comparative decline of their transcript levels could be observed during ripening, neither correlation between protein and mRNA. This suggests that protein abundance of these signaling components is likely controlled by post-translational modifications, like degradation, and not by a transcriptional regulation. Although SlETR1, SlETR2, and SlETR5 are the three least abundant ethylene receptors, it is possible that their higher protein levels in the mature green stage influence ethylene sensitivity by restraining ethylene signaling in this maturity stage due to their negative action. Their subsequent decrease in abundance during ripening could release this inhibitory action of ethylene sensitivity and perhaps eventually trigger fruit ripening. In this scenario, these receptors together with SlCTR2, could influence the initiation of tomato fruit ripening.

### EIN2 Levels Might Control Ethylene Sensitivity During Ripening

EIN2, on the other hand, is a positive regulator of ethylene signaling and is believed to play a central role in transmitting the ethylene signal from the ER to the nucleus (Alonso et al., 1999; Zheng and Zhu, 2016). Transgenic tomato plants in which *SlEIN2* expression is silenced, show a delayed fruit ripening phenotype, confirming that SlEIN2 is a positive regulator of ethylene signaling in tomato (Fu et al., 2005; Hu et al., 2010; Wang et al., 2016). We show now that SlEIN2 protein levels decreased directly in the breaker stage suggesting that ethylene sensitivity is gradually lost during fruit ripening. SlEIN2 protein abundance is directly correlated to *SlEIN2* expression, which also declines, but the drop became only significant in the red stage. Contrarily, Liu et al. (2015) reported, based on publicly available gene expression data, that *SlEIN2* expression did not change much during ripening, which does not match our findings using qPCR.

SlEIN2 is the largest protein analyzed in this work and in theory, based on the alignment with AtEIN2 (Supplementary Table 5), the C-terminal end of SlEIN2 could, given its size, also be present in the fractionated gel part. However, due to the microsomal membrane protein extraction used in this study, it is unlikely that the C-terminal cytosolic soluble portion was co-extracted with the membrane fraction, unless it had a strong membrane association. Therefore, what we can assure is the quantification of the complete protein SlEIN2, but not of its C-terminal portion, which anyway would be present in a lower percentage than the intact SlEIN2 protein. The fact that the quantification was mainly of the intact protein would mean that SlEIN2 levels are declining during ripening, possible through the ETP-mediated degradation (Qiao et al., 2009). This would explain why the decrease in the protein levels could already be seen in the breaker stage, while for the gene expression, levels became only significant at the red stage. Hence, the apparent discrepancy between the more constant transcription levels and the falling protein levels of SlEIN2. The discovery of the exact cleavage site of SlEIN2 in tomato, as well as the retirement of additional peptides that are exclusively located in the N-terminal part, would allow us to distinguish the abundance of both the N- and C-terminal part of EIN2, and give more insight in the regulatory dynamics of this enigmatic protein.

## Conclusion

This work describes a feasible and reproducible technique to identify and quantify the low abundant ethylene signaling proteins ethylene receptors (ETRs), CTRs and EIN2 in tomato pericarp. The strategy is composed of (i) microsomal membrane extraction, (ii) fractionation of the protein sample through 1-D gel, (iii) in-gel tryptic digestion and (iv) identification and absolute quantification through the monitoring of several unique peptides of the target proteins by PRM. The combined quantification of protein and mRNA levels of the ethylene signaling components during ripening has revealed different patterns between gene expression and protein abundance which might collectively modulate and control ethylene sensitivity and thus the timing and rate of fruit ripening. Our hypothesis is that some receptors would largely control the ethylene sensitivity and, therefore, the ripening process, like SlETR3 and SlETR4 with the help of SlCTR1, some of the most abundant proteins, and possibly SlCTR4. Other signaling components such as SlETR6, SlETR7, and SlCTR3 show an unaltered protein abundance during the onset of ripening and might therefore be important to sustain the ripening process. Finally, proteins such as SlETR1, SlETR2, SlETR5, and SlCTR2 show a rapid decline in protein abundance, which might suggest that they could control the initiation of ripening. SlEIN2, being a positive regulator of ethylene signaling, also show a declining abundance profile, and could therefore also control ethylene sensitivity during climacteric fruit ripening of tomato. In conclusion, it seems that ethylene sensitivity is differently controlled by a balanced turnover of the different components of the ethylene signaling pathway, combining positive and negative feedback regulations.

Future mass spectrometry analyses are needed to reveal the specific proteolytic cleavage site of SlEIN2 and to study the phosphorylation dynamics of both the receptors and SlEIN2 during ripening. Finally, a broad quantitative proteomics study including additional downstream signaling transcription factors such as the EILs and ERFs could help us to better understand ethylene sensitivity and signaling during climacteric fruit ripening of tomato.

## Author Contributions

CM, BF, HP, MH, and KL designed the experiments. CM carried out the experiments, data analysis, prepared the figures, and wrote the manuscript. CM, BF, HP, MH, GVR, GB, BVdP, KL, and BN helped to improve the manuscript and participated in discussions. All authors provided feedback on the manuscript and gave their final approval for submission.

## Conflict of Interest Statement

The authors declare that the research was conducted in the absence of any commercial or financial relationships that could be construed as a potential conflict of interest.

## Footnotes

1 http://bioinfo.ut.ee/primer3/

## Supplementary Material

Figure S1 Dilution curves for the PRM analysis of 0–200 fmol/μL of the selected heavy labeled peptides for the proteins SlETR1-SlETR7, SlCTR1-SlCTR3 and SlEIN2 and linearity expressed by coefficient of determination (R2).

Figure S2 Absolute quantification (fmol of target protein/μg of total membrane proteins) of the peptides of SlETR1-SlETR7, SlCTR1-SlCTR3 and SlEIN2 during tomato fruit ripening. MG, mature green; BR, breaker; OR, orange; R, red tomatoes. Error bars represent the standard error of the mean based on six biological replicates. Different uppercase letters indicate significant differences between the absolute protein concentration levels of the four tomato ripening stages determined by Tukey’s Honestly Significant Difference (HSD) test (*P* < 0.05).

Figure S3 Correlation between protein and gene expression levels of the SlETR1-SlETR7, SlCTR1-SlCTR3 and SlEIN2. Significant correlations are represented with an asterisk in the chart title and non-significant correlation with the letters N.S. The protein, gene expression data and their standard errors were normalized for visualization.

Table S1 List of unlabeled peptides tested for the assay development. The ones marked in yellow were the identified and quantified peptides (labeled peptides were order afterwards for these ones). The ones in green were promisingly identified but labeled peptide for them were not obtained.

Table S2 Selected results output of the use of the mProphet algorithm of the targeted peptide identifications trained with the second best peak option.

Table S3 RT-qPCR primers for the 12 ethylene signaling and 3 reference genes used in this study. Primers were designed with the Primer3 web tool (http://bioinfo.ut.ee/primer3/). Primer specificity was checked by BLAST-ing against all tomato EST’s and known cDNA sequences.

Table S4 Amino acid sequences of the proteins SlETR1-SlETR7, SlCTR1-SlCTR3 and SlEIN2 obtained from Uniprot (Bateman et al., 2015). Their Uniprotannotated transmembrane domains are underlined, their possible phosphorylation sites are highlighted in green and their GAF domain, kinase domain and response regulatory domains are represented in green, blue and orange fonts, respectively. The peptides used for the quantifications of the proteins in the current study are highlighted in yellow.

Table S5 Cluster alignment of Q9S814 (AtEIN2) and Q6Q2C1 (SlEIN2) with the alignment tool of Uniprot. The C-terminal part of AtEIN2 is highlighted in green based on the results of Qiao et al. (2012). The two peptides of SlEIN2 identified and quantified in this work are highlighted in yellow.
