# Supplementary material for: Ethylene Receptors, CTRs and EIN2 Target Protein Identification and Quantification Through Parallel Reaction Monitoring During Tomato Fruit Ripening
Source: Front Plant Sci. 2018 Nov 8;9:1626. doi: 10.3389/fpls.2018.01626 (PMC6235968; doi:10.3389/fpls.2018.01626)
Supplement: Figure S1 — Dilution curves for the PRM analysis of 0–200 fmol/μL of the selected heavy labeled peptides for the proteins SlETR1-SlETR7, SlCTR1-SlCTR3 and SlEIN2 and linearity expressed by coefficient of determination (R2). [file Presentation_1.pptx]

## Slide 1
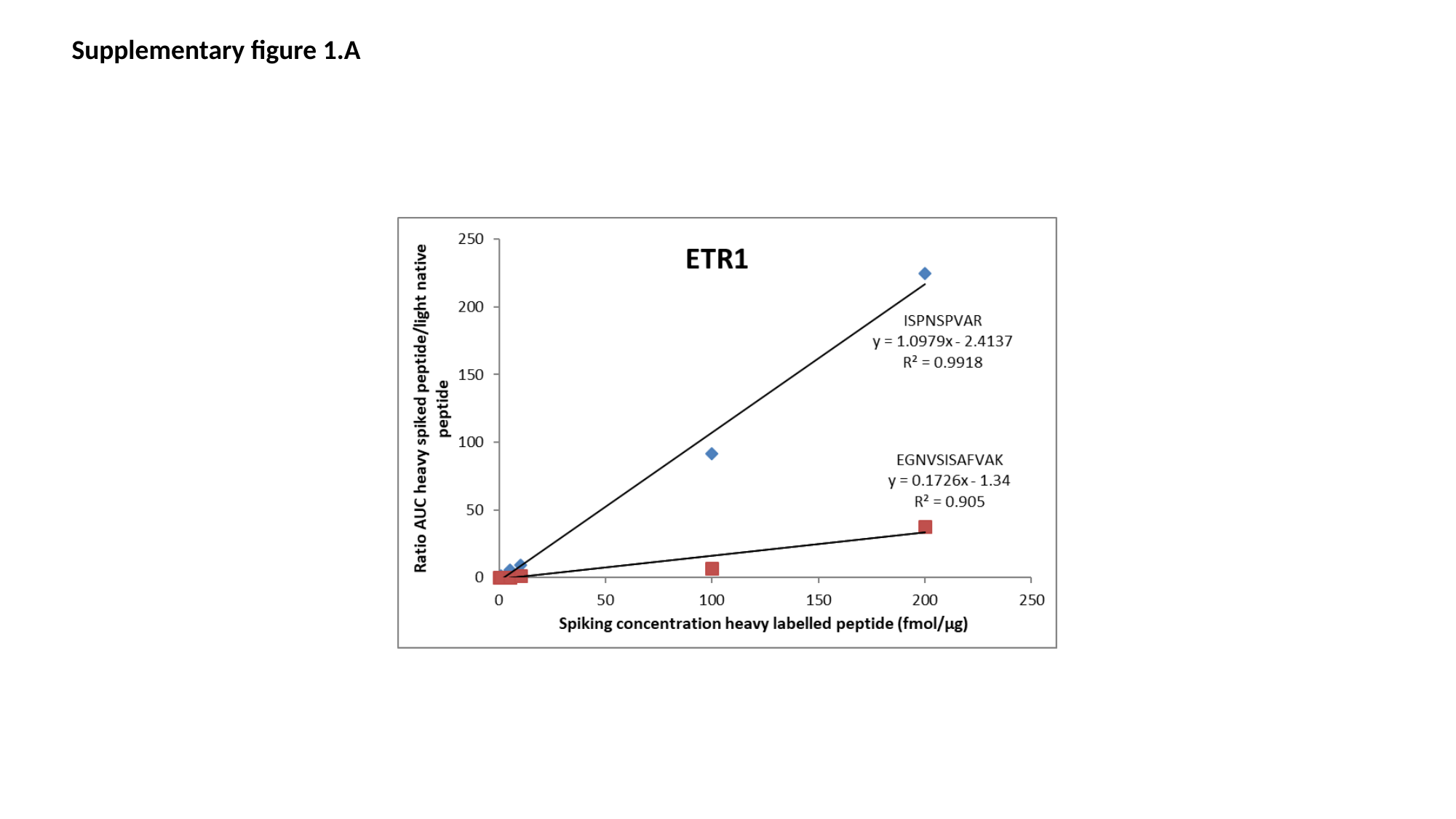

Supplementary figure 1.A

## Slide 2
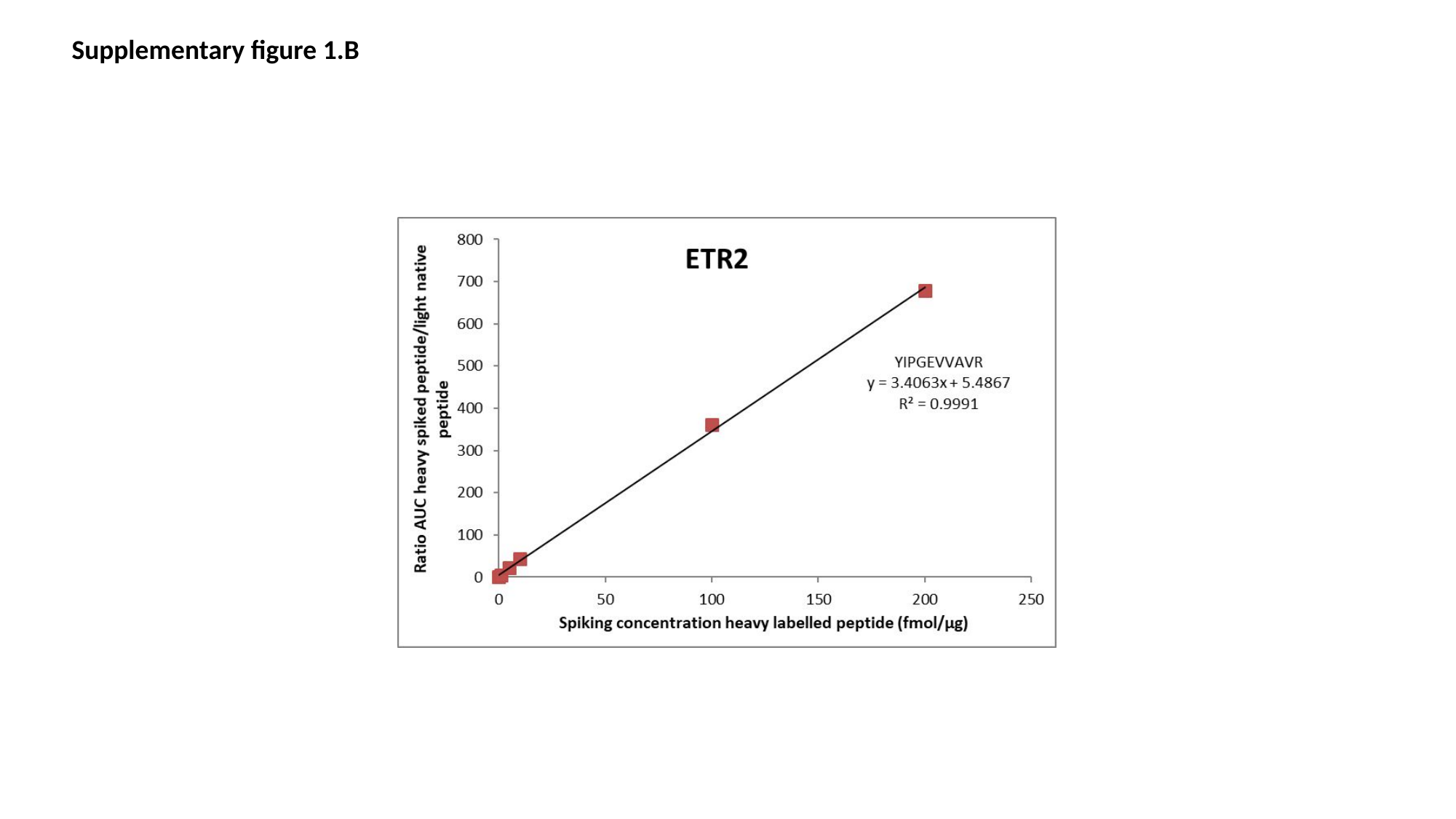

Supplementary figure 1.B

## Slide 3
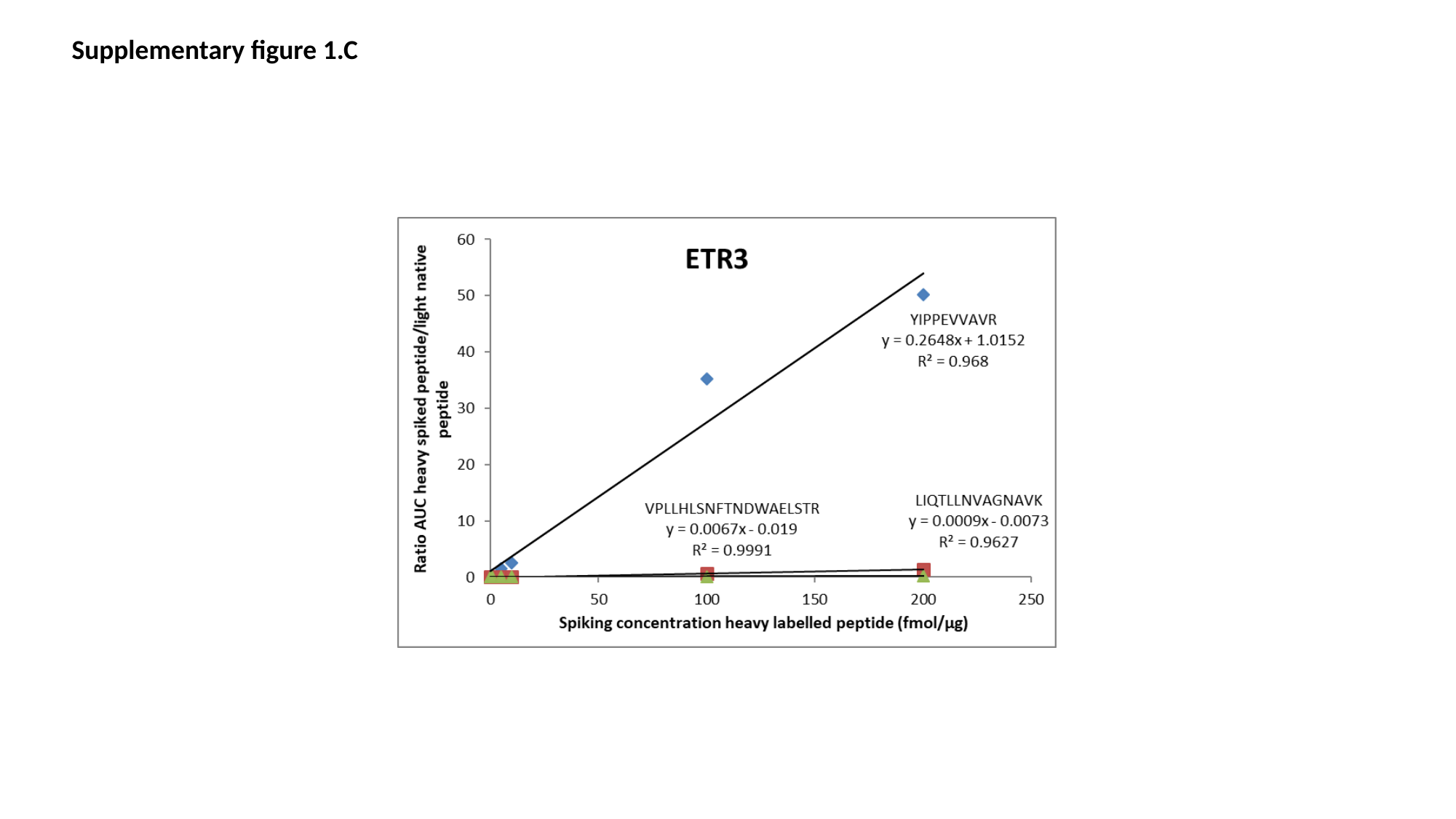

Supplementary figure 1.C

## Slide 4
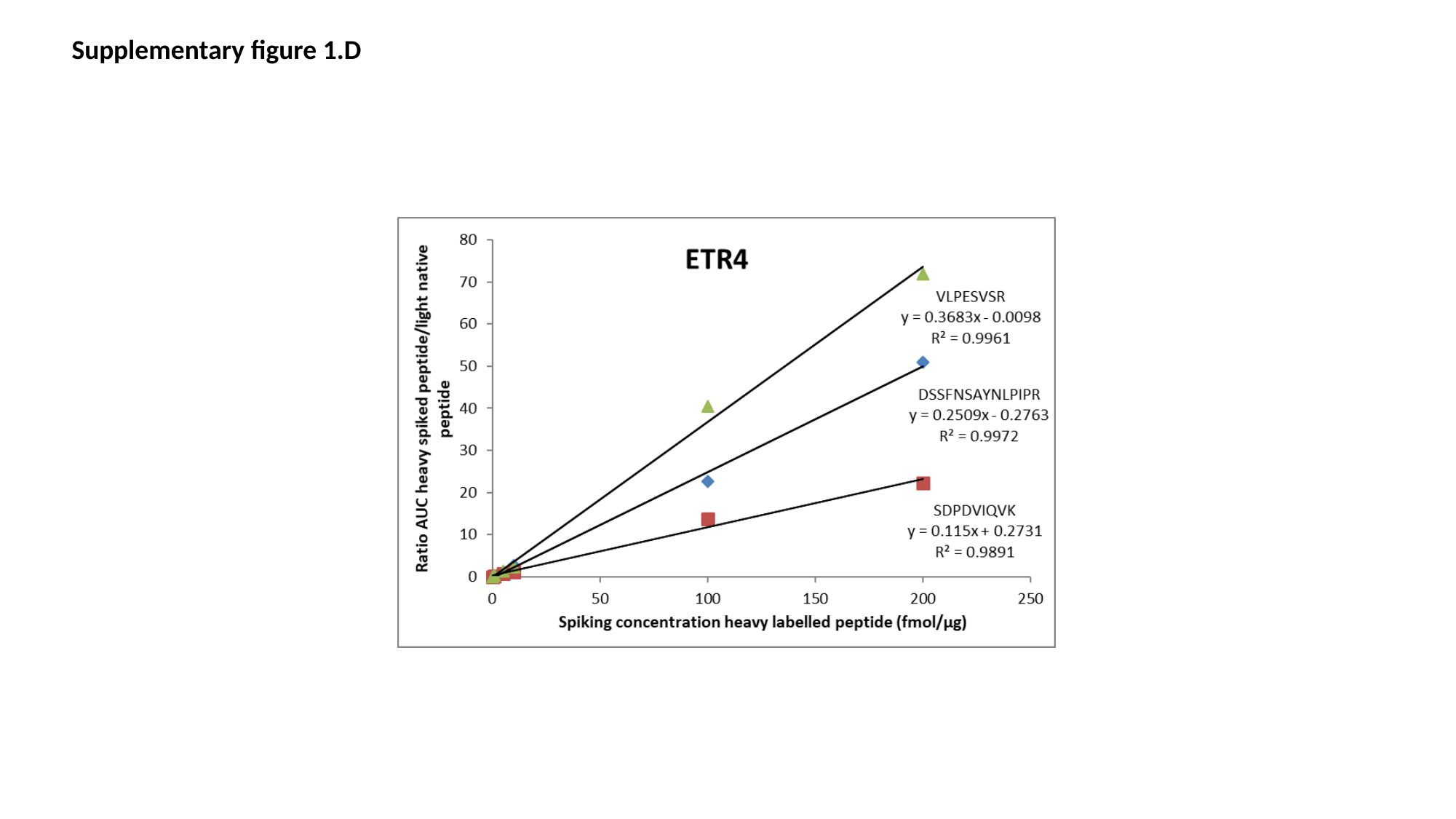

Supplementary figure 1.D

## Slide 5
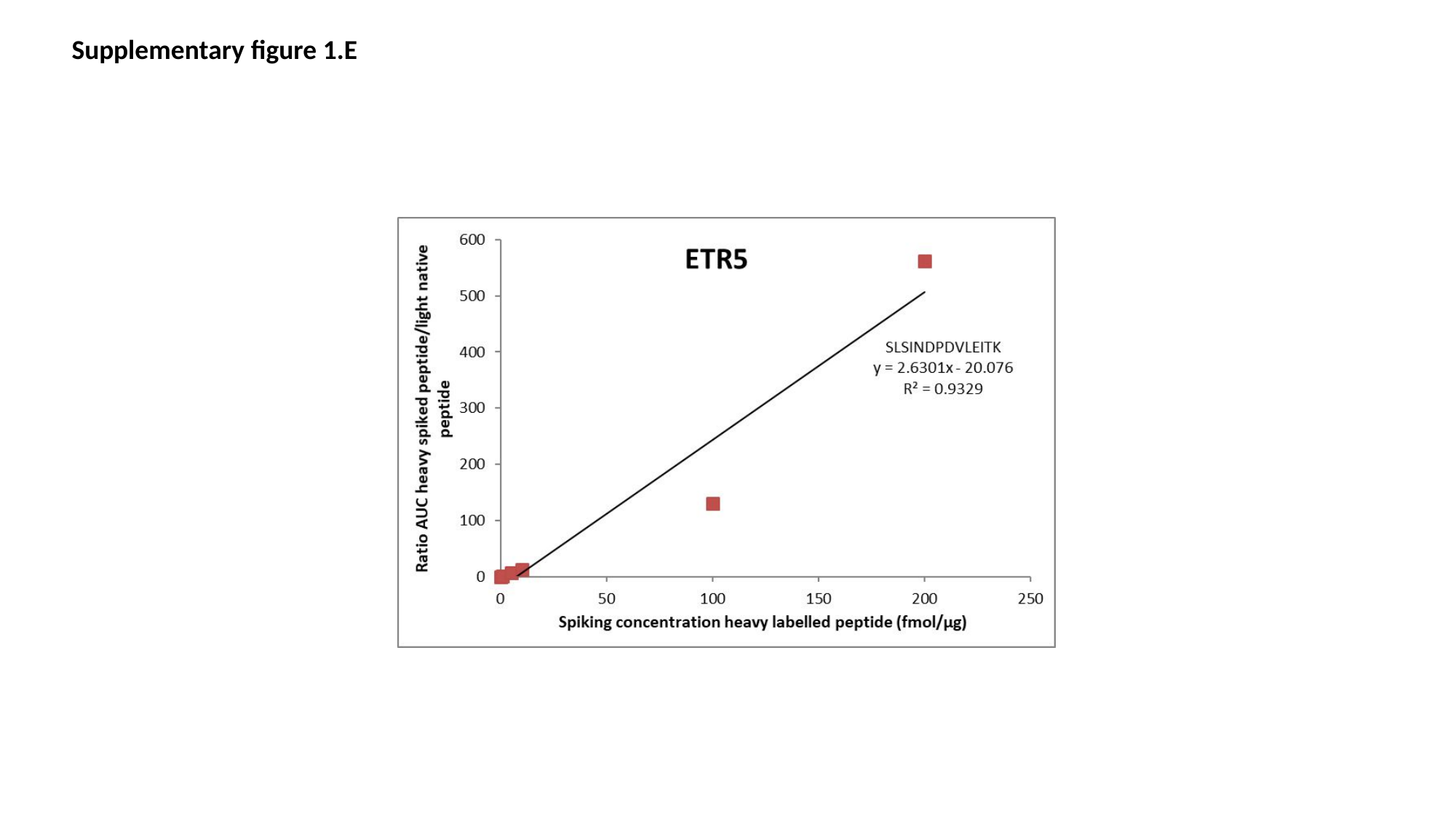

Supplementary figure 1.E

## Slide 6
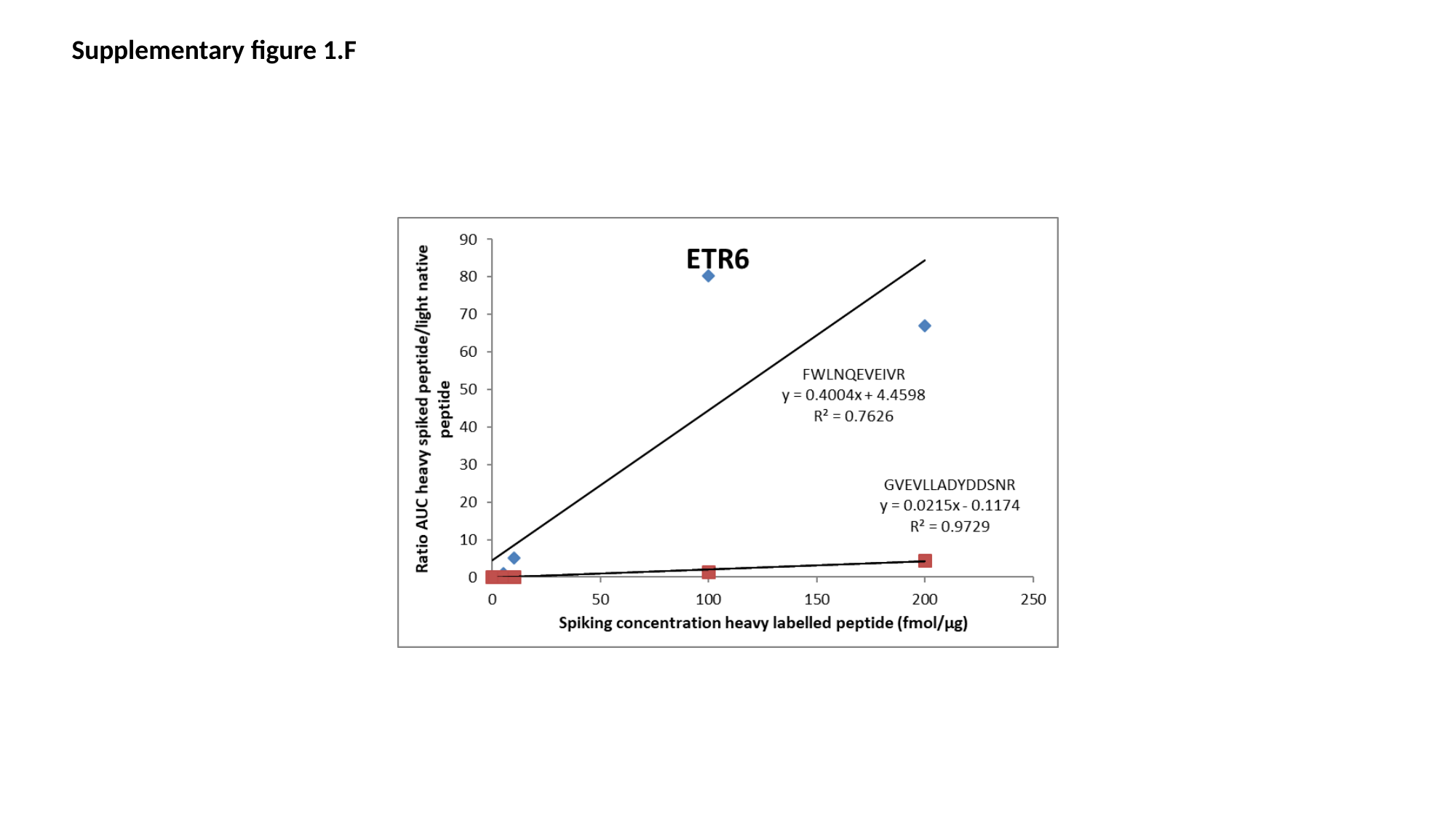

Supplementary figure 1.F

## Slide 7
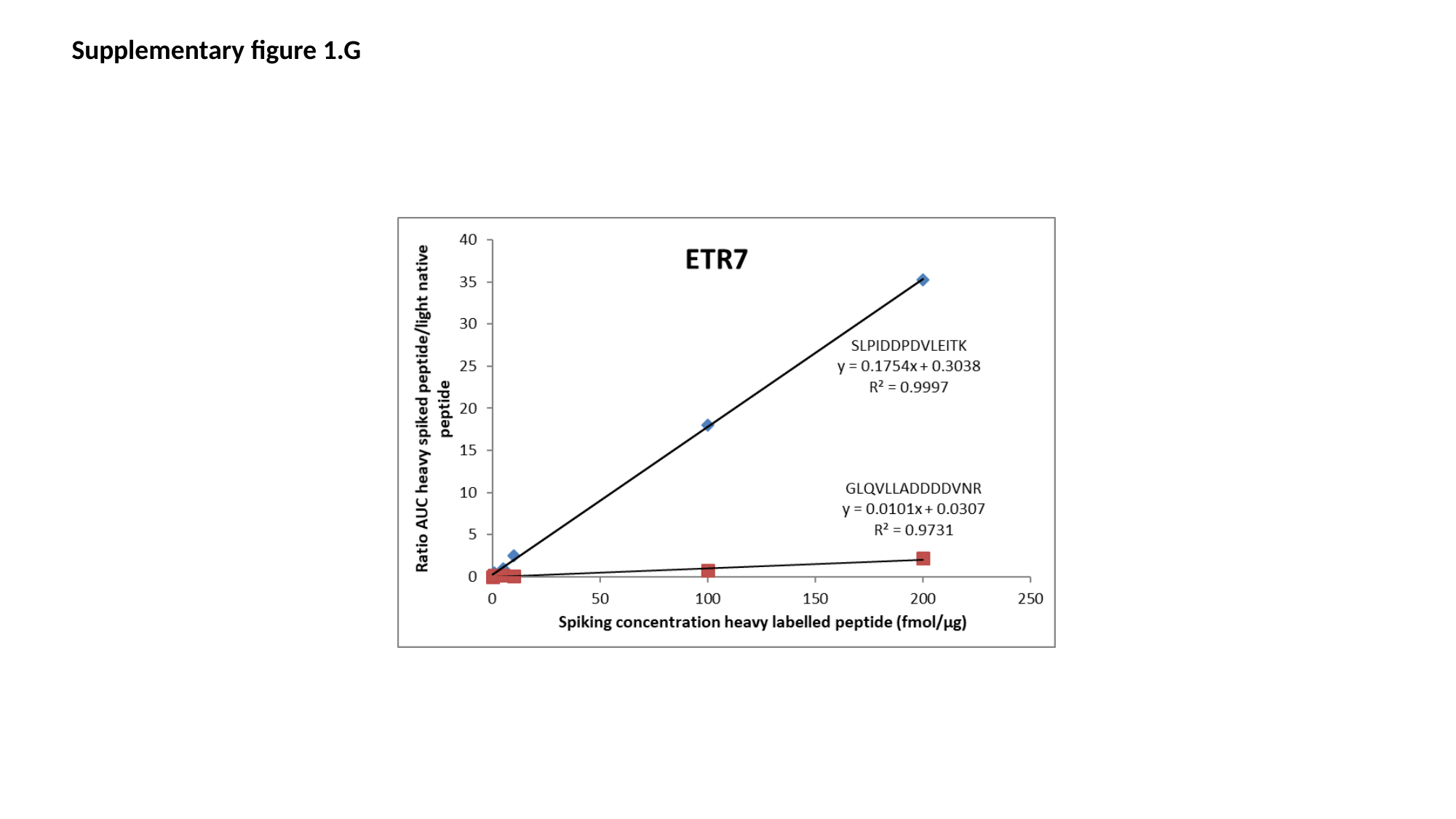

Supplementary figure 1.G

## Slide 8
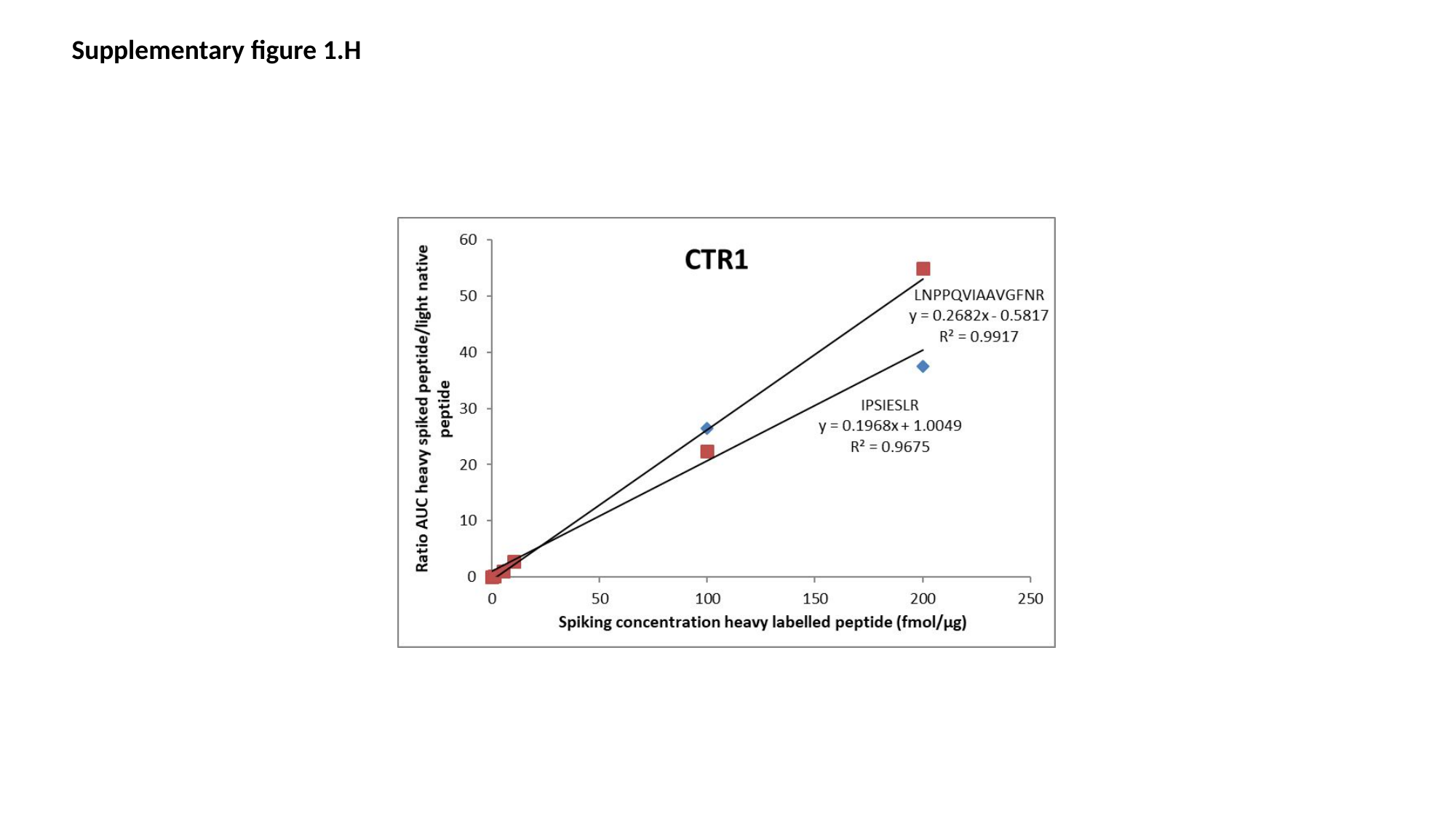

Supplementary figure 1.H

## Slide 9
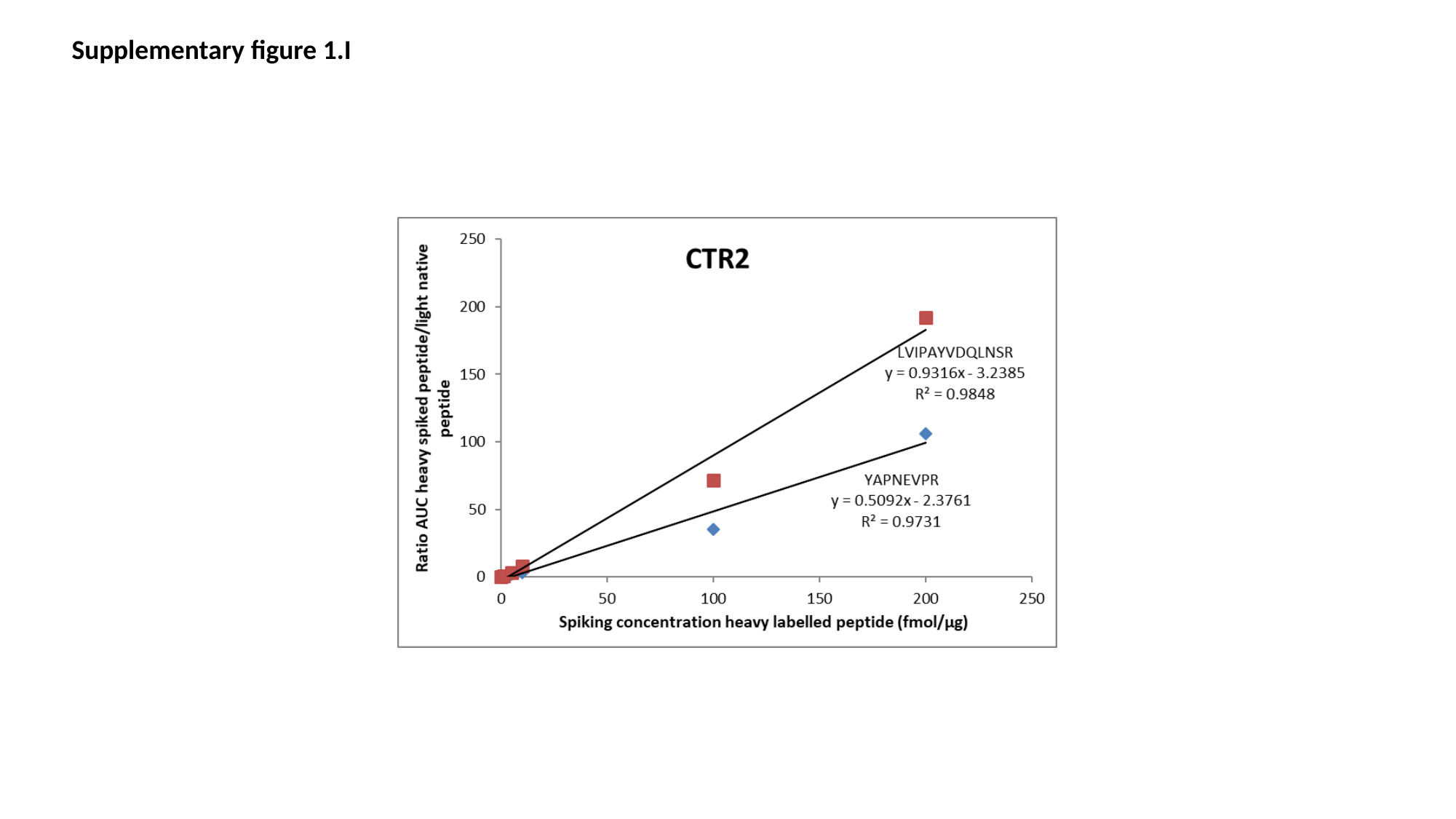

Supplementary figure 1.I

## Slide 10
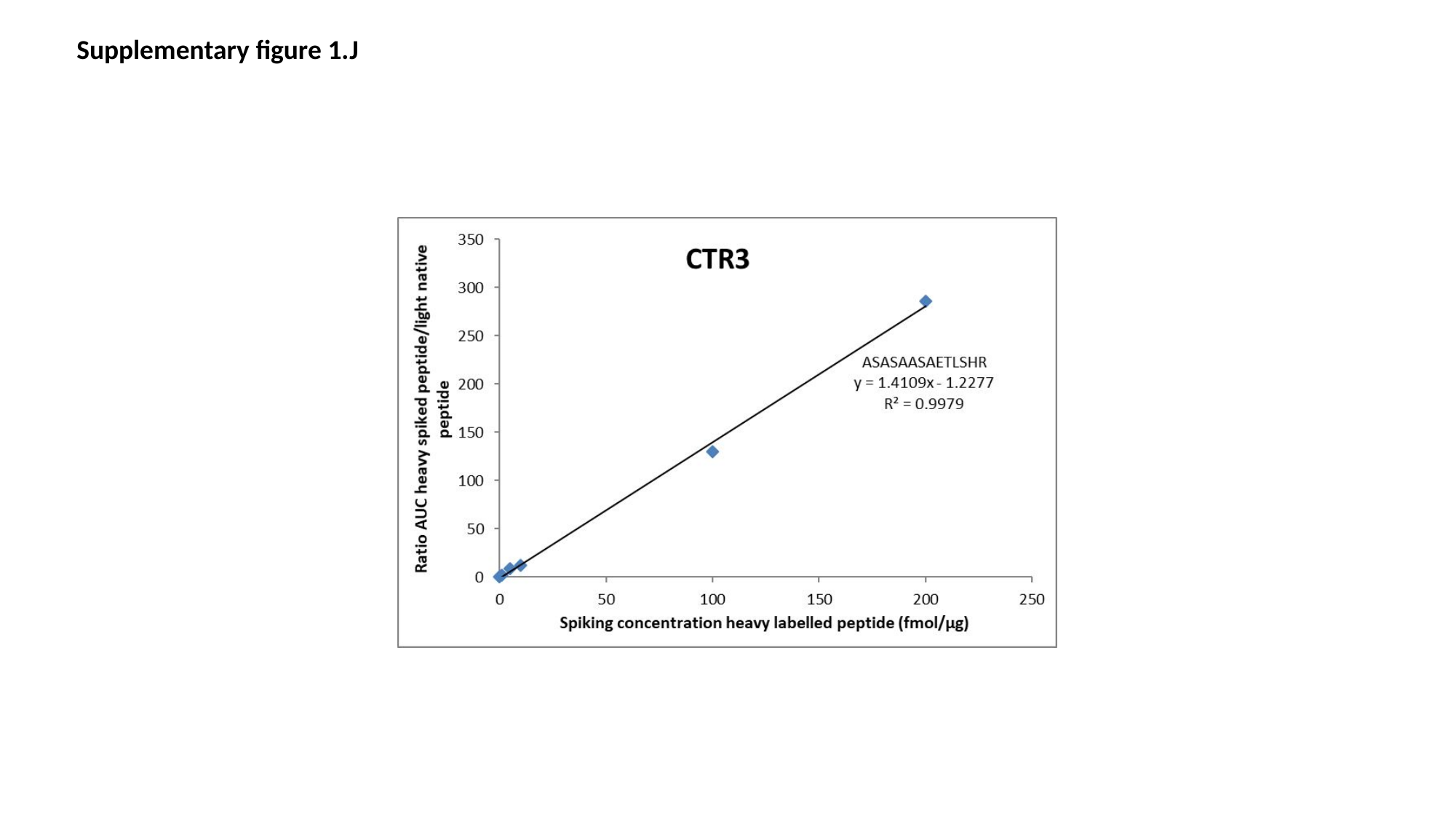

Supplementary figure 1.J

## Slide 11
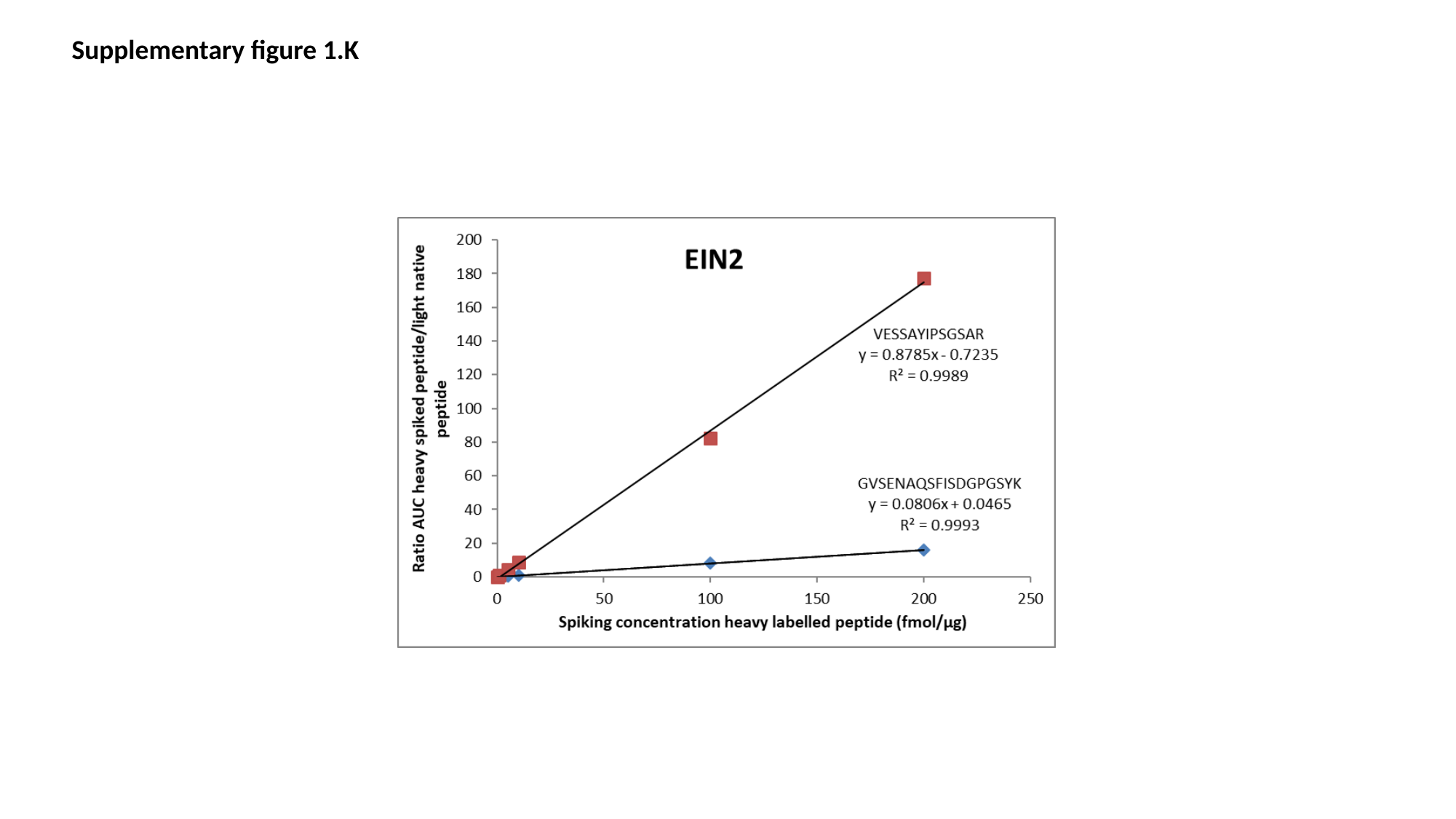

Supplementary figure 1.K
