# Supplementary material for: Ethylene Receptors, CTRs and EIN2 Target Protein Identification and Quantification Through Parallel Reaction Monitoring During Tomato Fruit Ripening
Source: Front Plant Sci. 2018 Nov 8;9:1626. doi: 10.3389/fpls.2018.01626 (PMC6235968; doi:10.3389/fpls.2018.01626)
Supplement: Figure S2 — Absolute quantification (fmol of target protein/μg of total membrane proteins) of the peptides of SlETR1-SlETR7, SlCTR1-SlCTR3 and SlEIN2 during tomato fruit ripening. MG, mature green; BR, breaker; OR, orange; R, red tomatoes. Error bars represent the standard error of the mean based on six biological replicates. Different uppercase letters indicate significant differences between the absolute protein concentration levels of the four tomato ripening stages determined by Tukey’s Honestly Significant Difference (HSD) test (P < 0.05). [file Presentation_2.PPTX]

## Slide 1
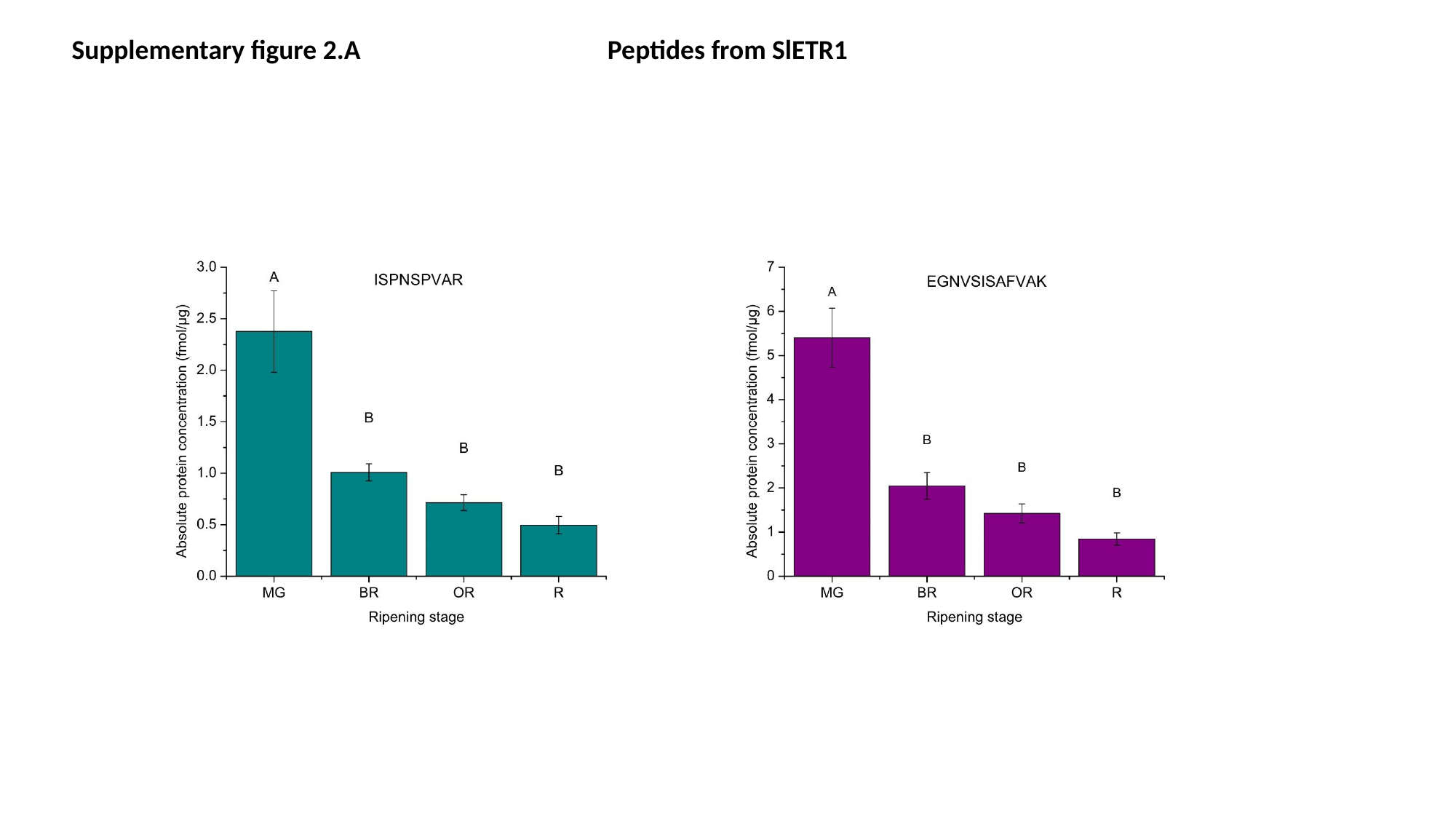

Supplementary figure 2.A
Peptides from SlETR1

## Slide 2
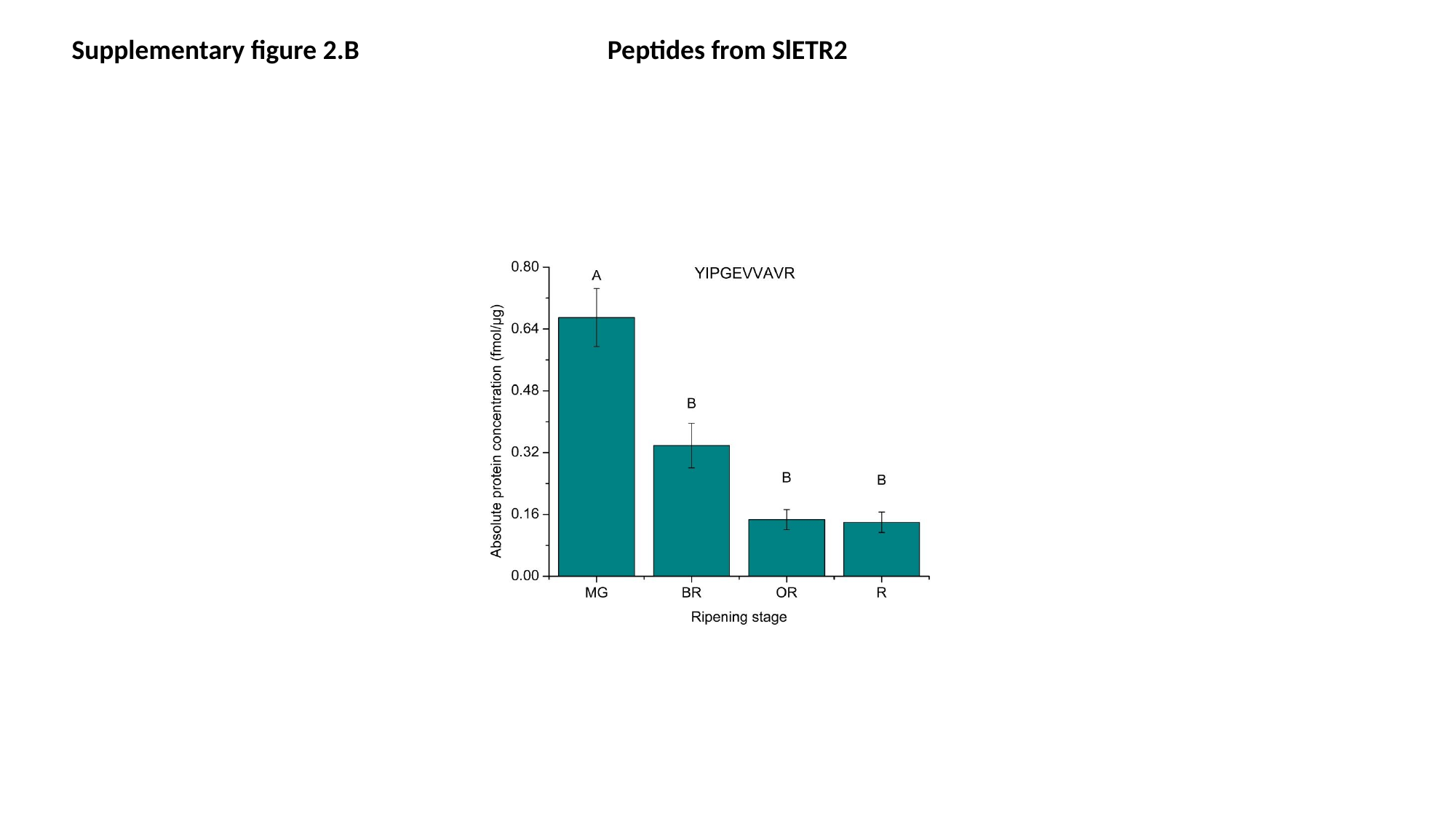

Supplementary figure 2.B
Peptides from SlETR2

## Slide 3
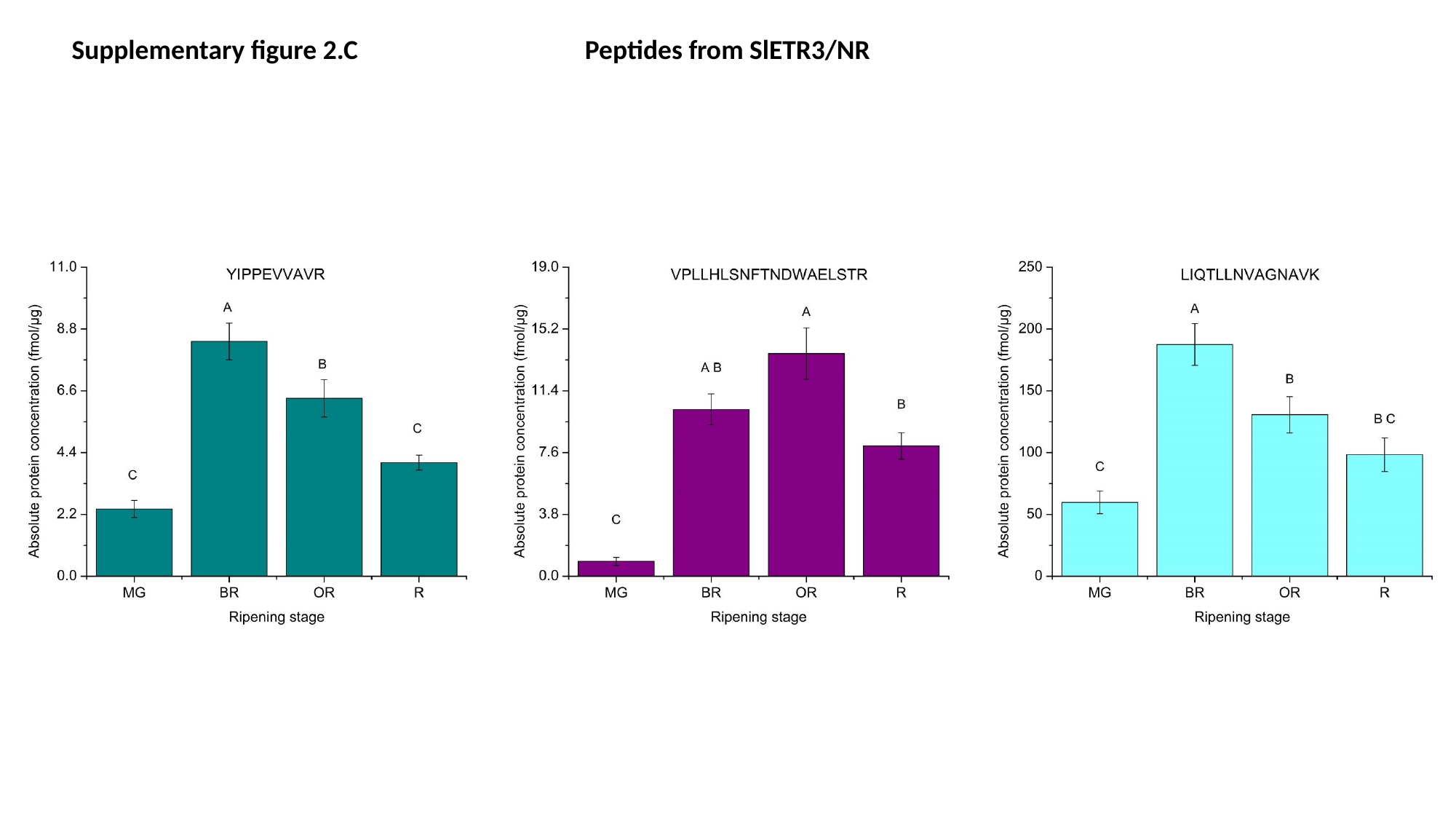

Supplementary figure 2.C
Peptides from SlETR3/NR

## Slide 4
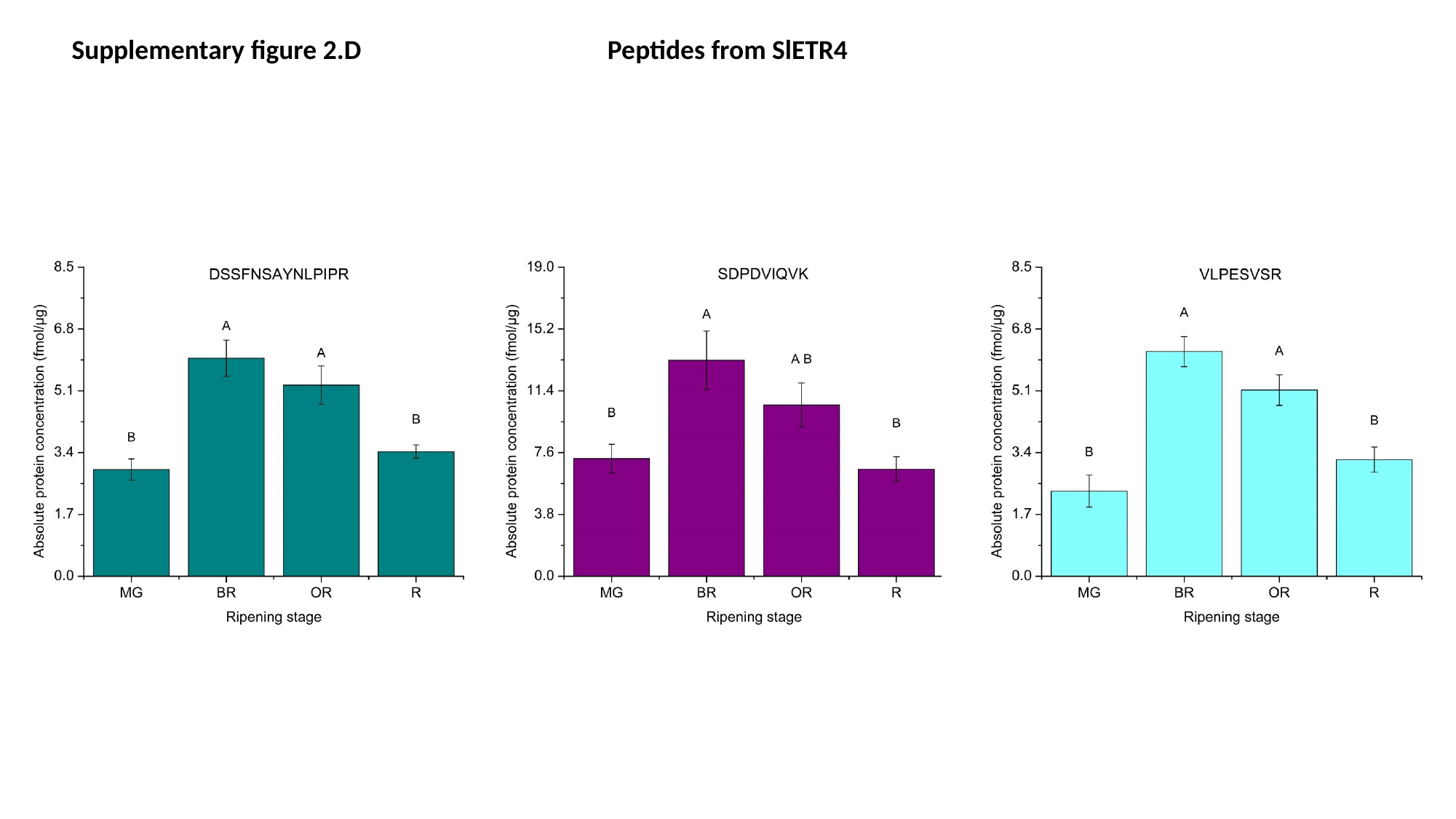

Supplementary figure 2.D
Peptides from SlETR4

## Slide 5
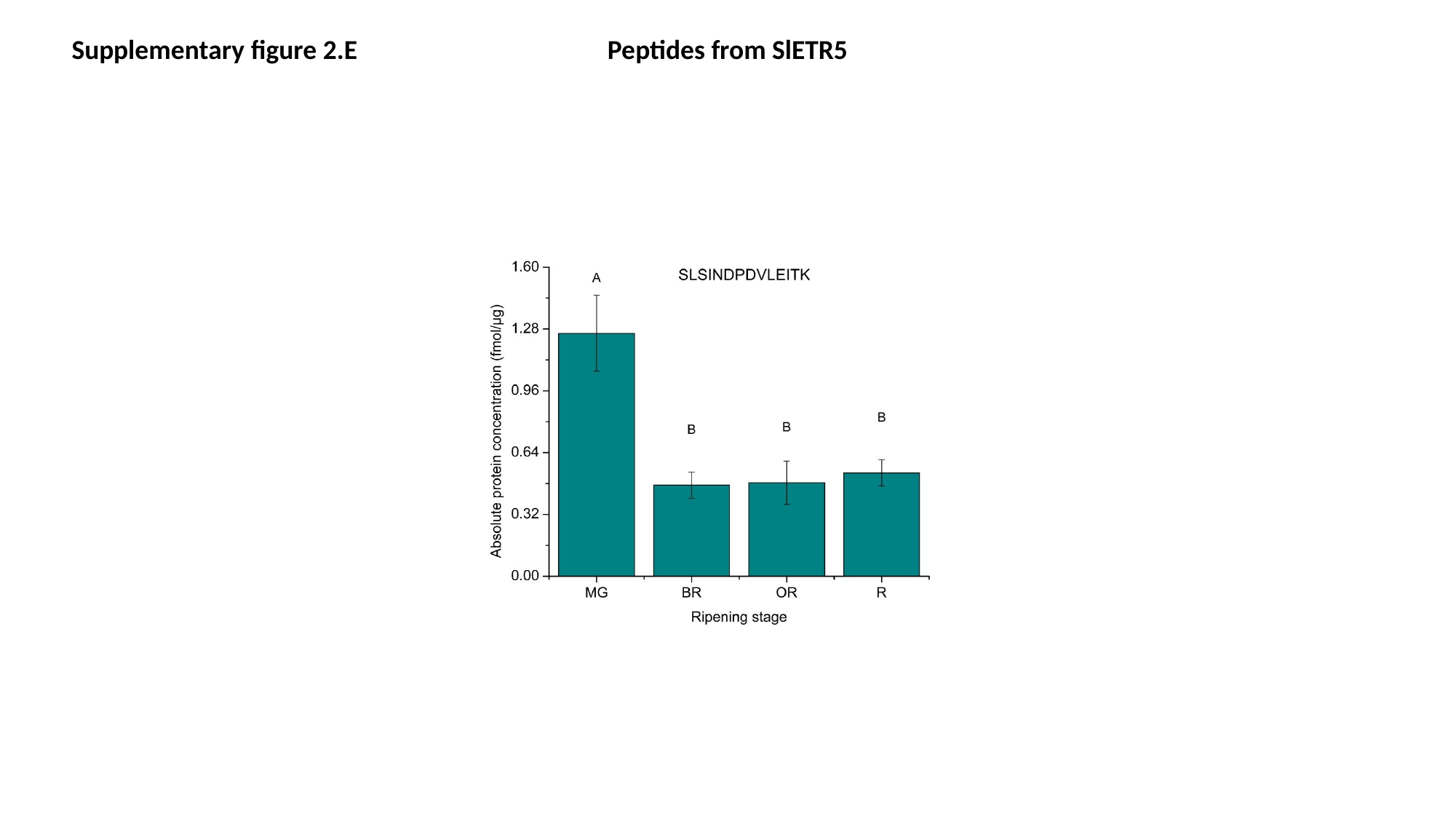

Supplementary figure 2.E
Peptides from SlETR5

## Slide 6
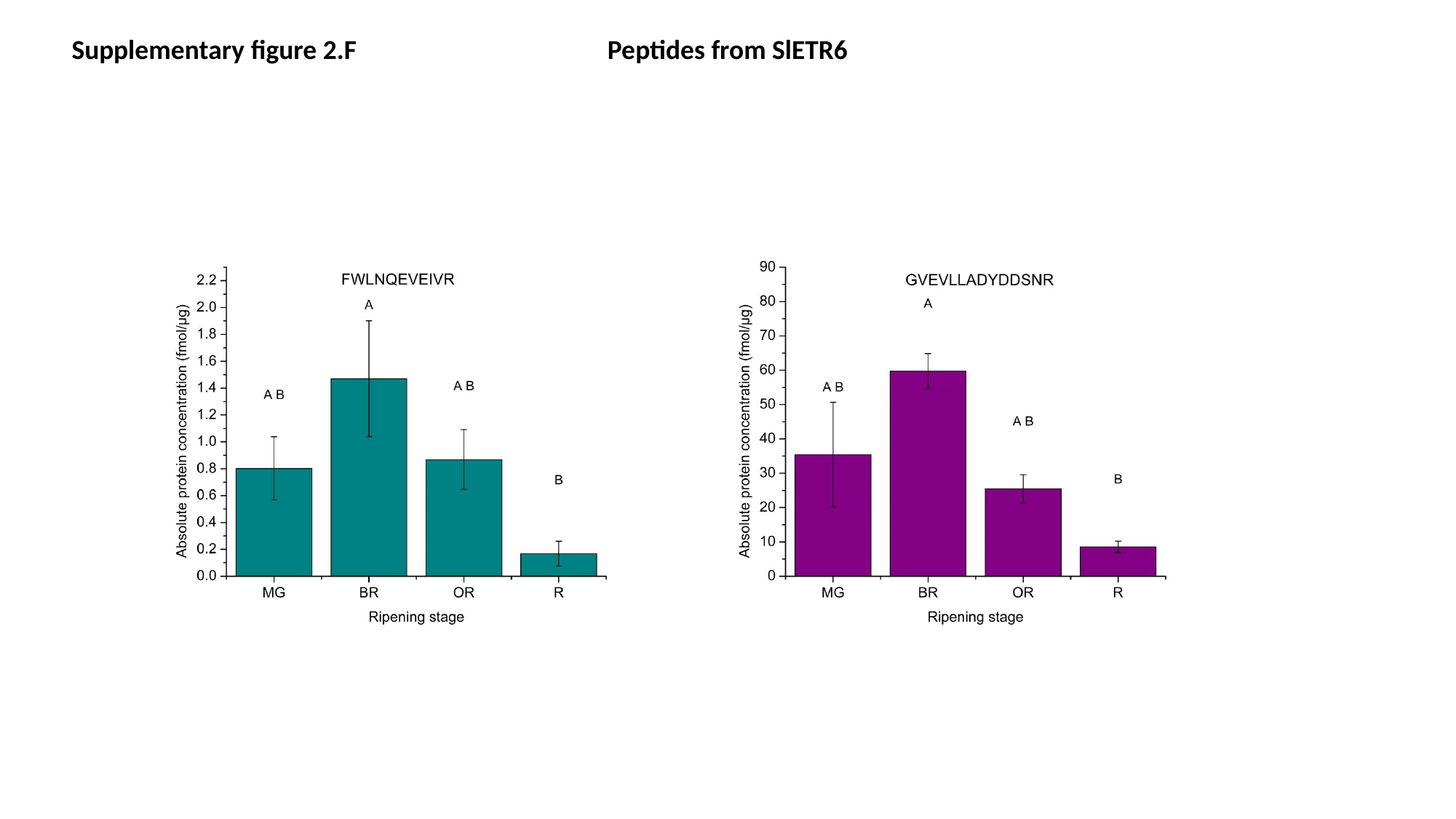

Supplementary figure 2.F
Peptides from SlETR6

## Slide 7
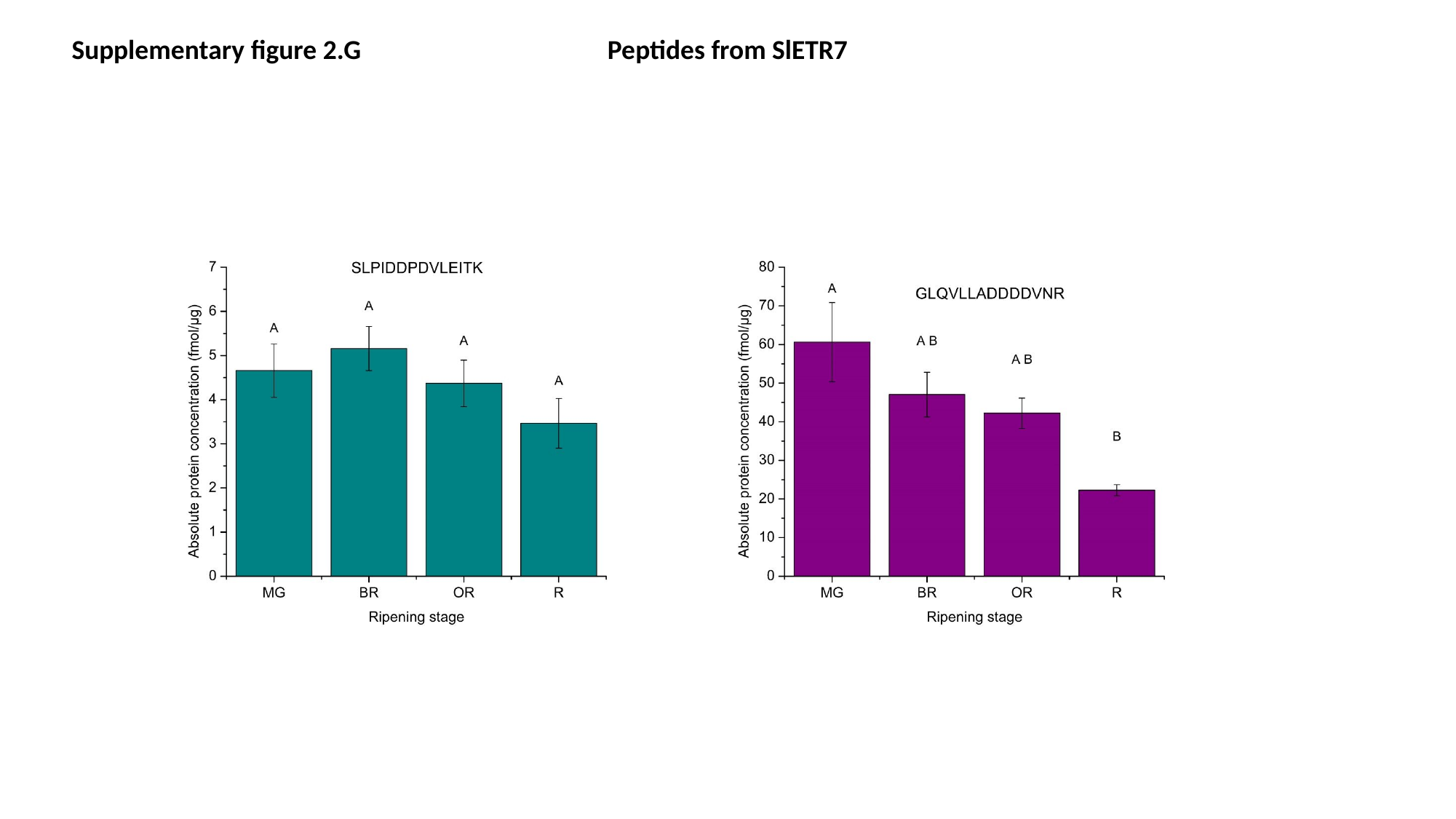

Supplementary figure 2.G
Peptides from SlETR7

## Slide 8
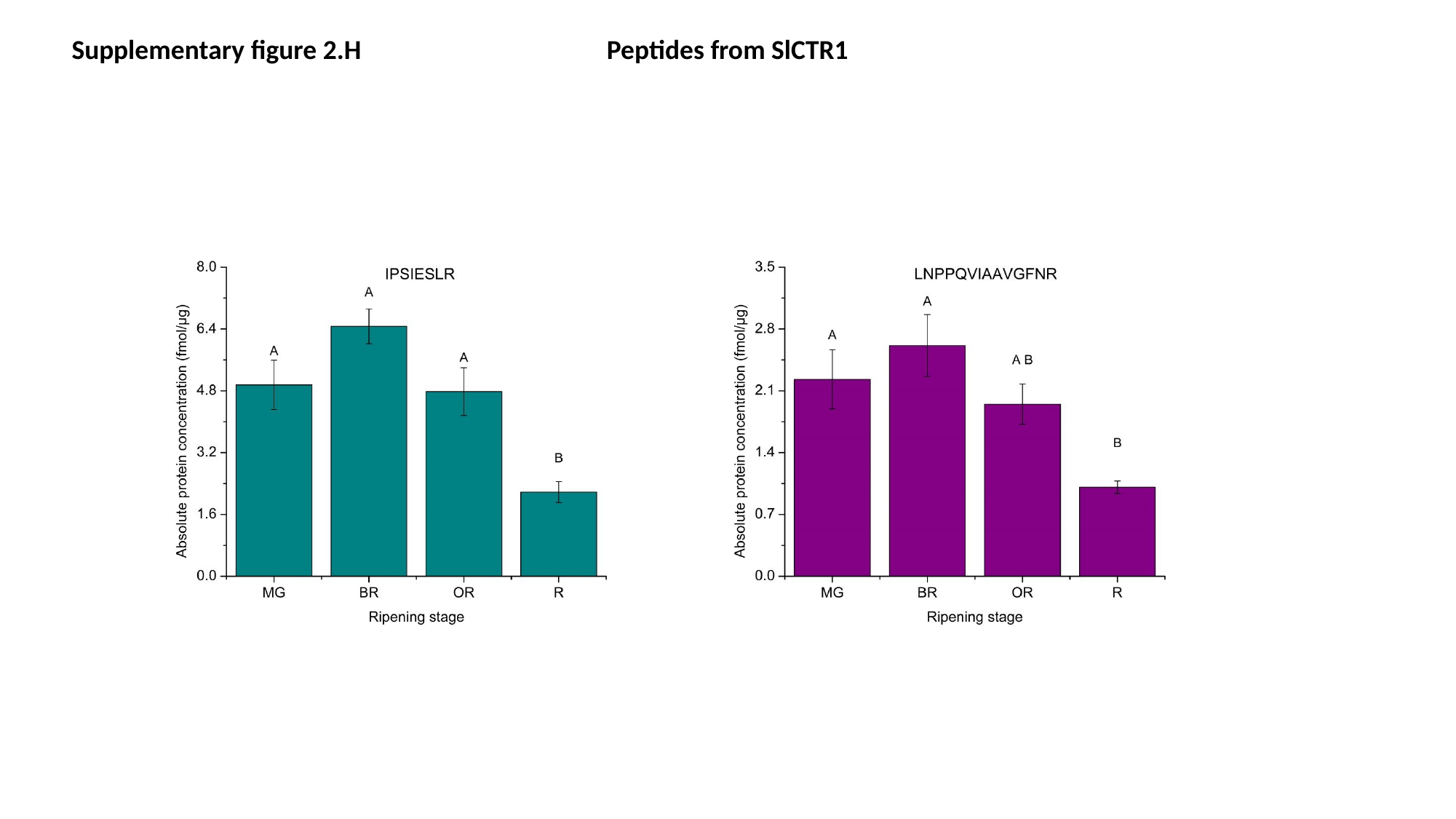

Supplementary figure 2.H
Peptides from SlCTR1

## Slide 9
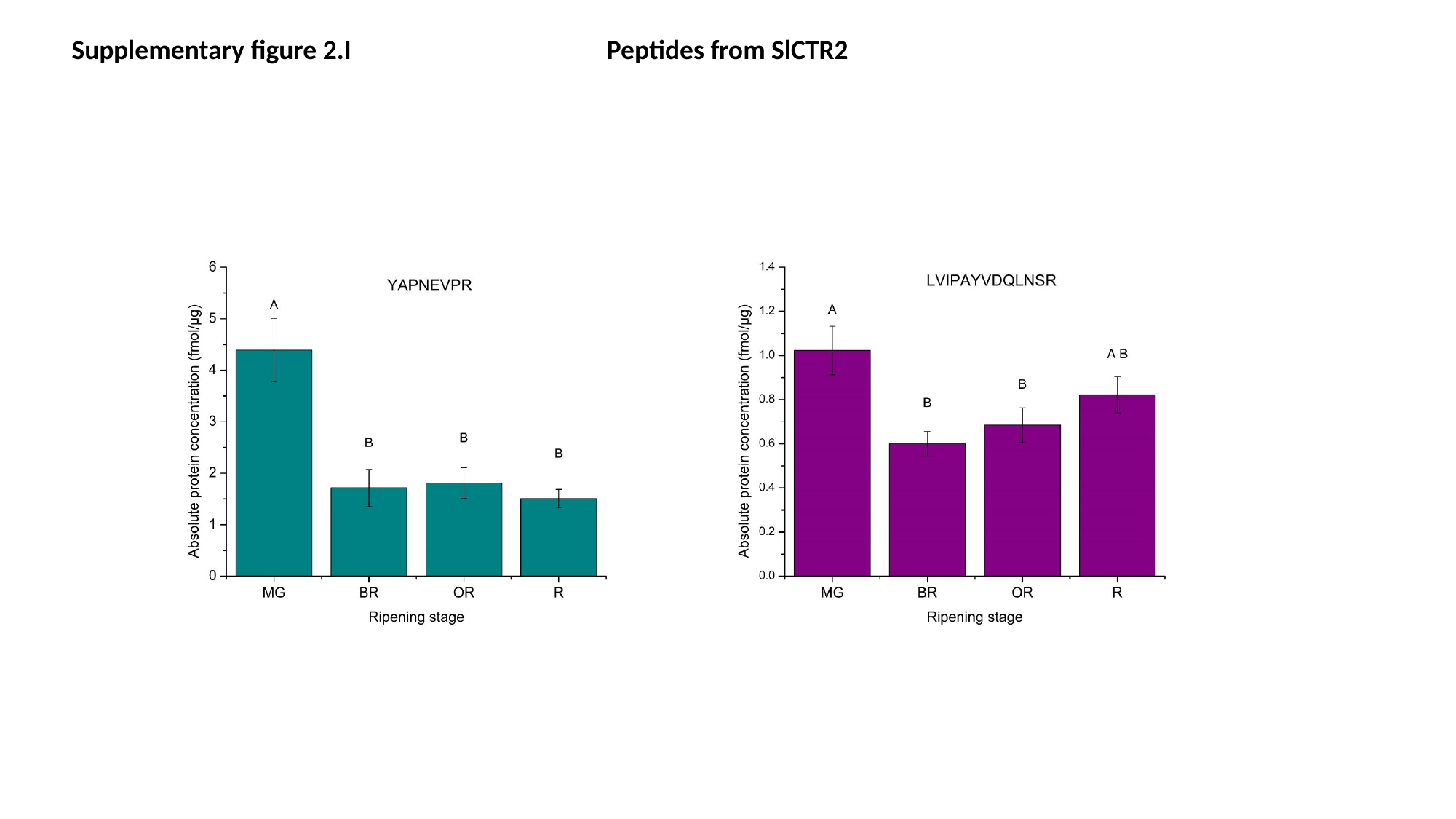

Supplementary figure 2.I
Peptides from SlCTR2

## Slide 10
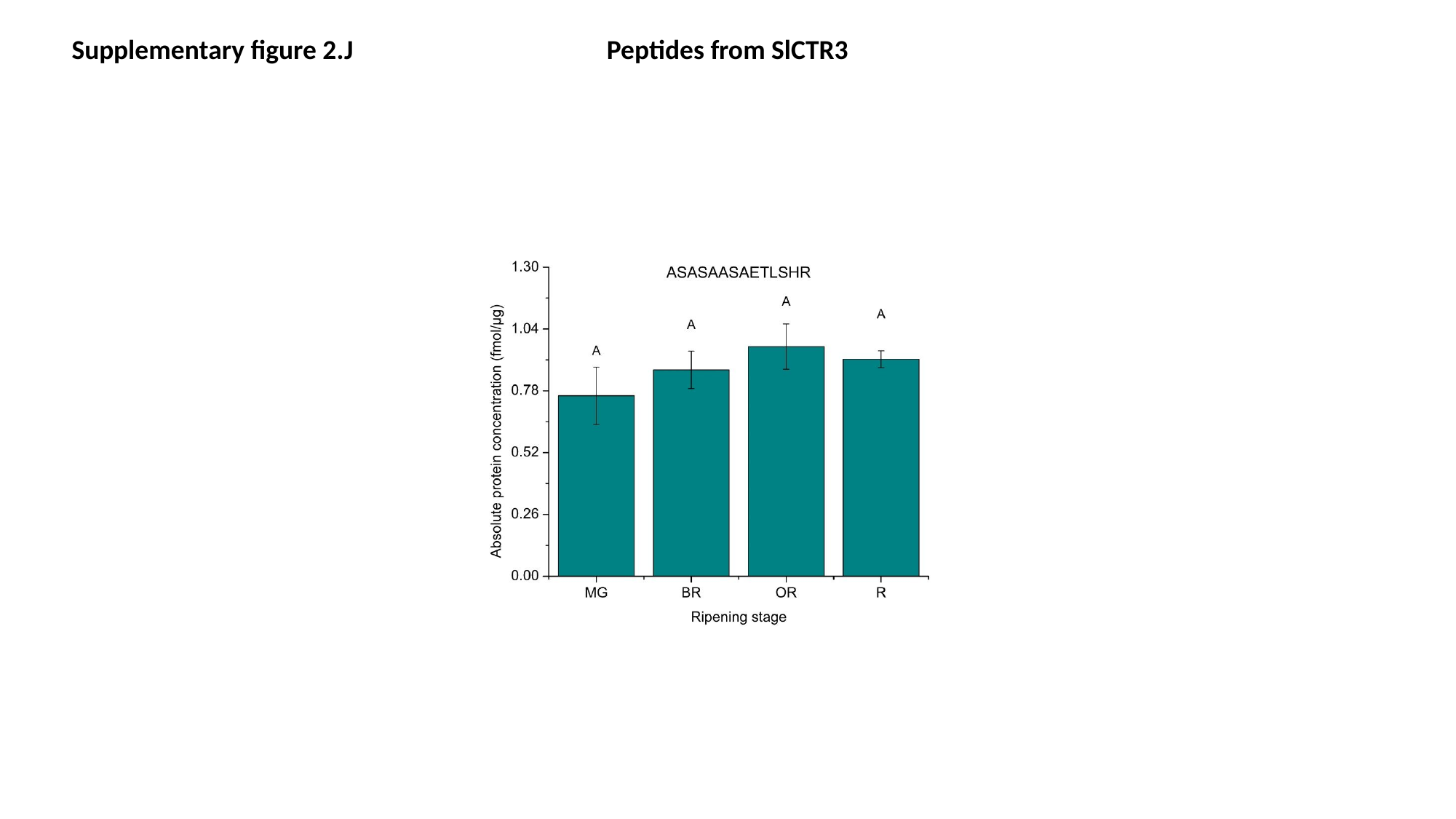

Supplementary figure 2.J
Peptides from SlCTR3

## Slide 11
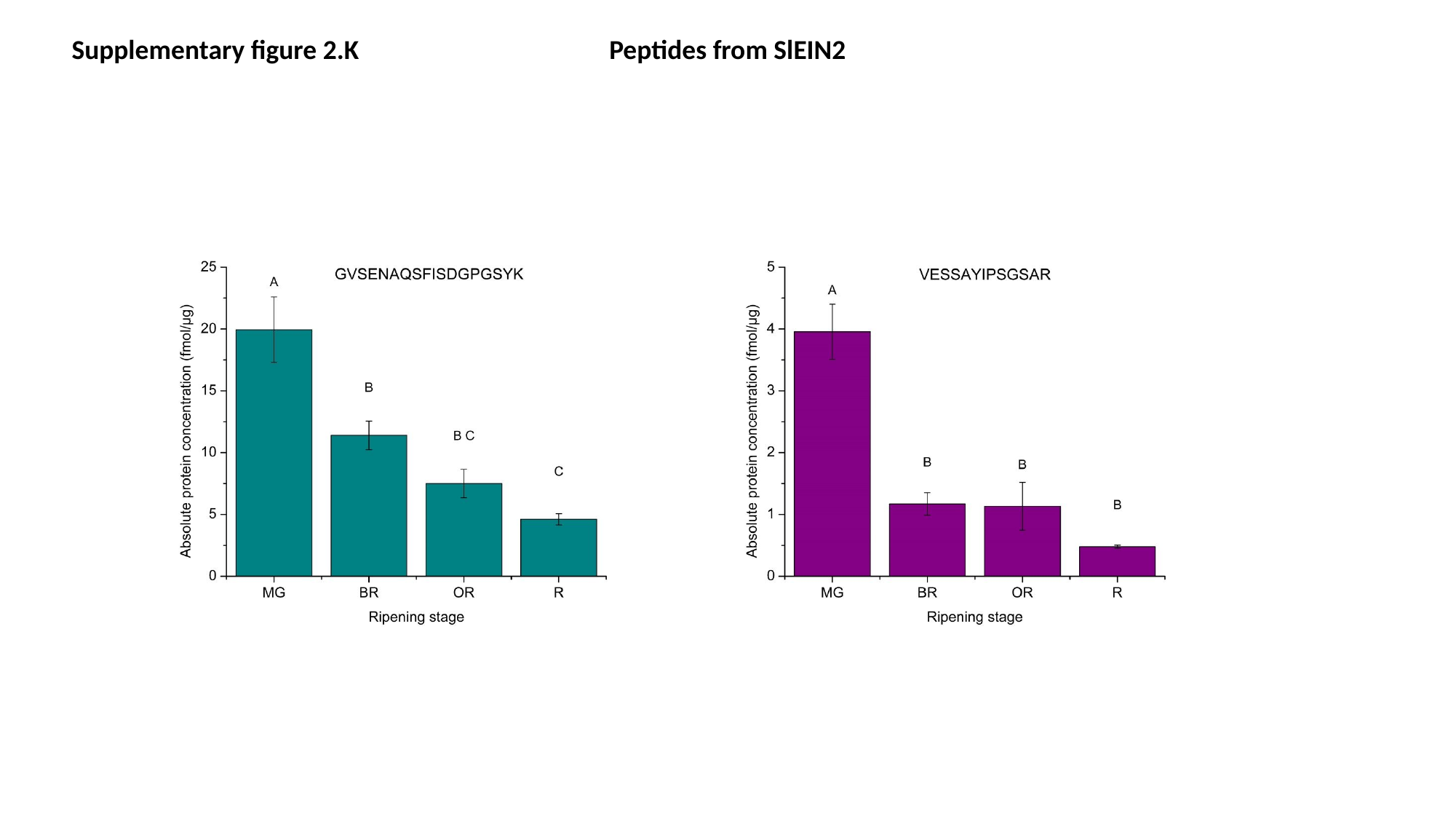

Supplementary figure 2.K
Peptides from SlEIN2
