# Supplementary figures and images for: Ethylene Receptors, CTRs and EIN2 Target Protein Identification and Quantification Through Parallel Reaction Monitoring During Tomato Fruit Ripening
Source: Front Plant Sci. 2018 Nov 8;9:1626. doi: 10.3389/fpls.2018.01626 (PMC6235968; doi:10.3389/fpls.2018.01626)

## Slide 1
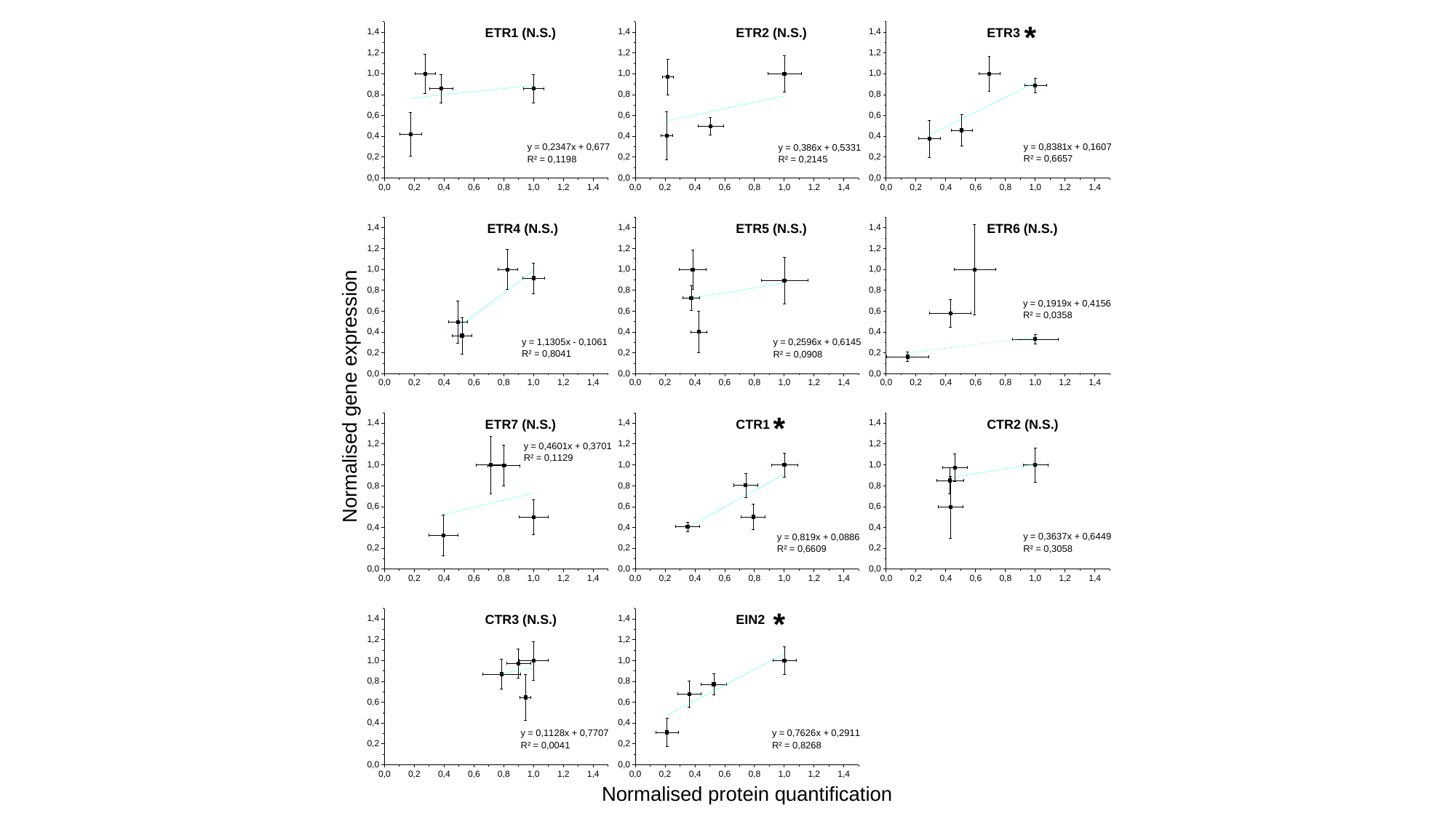

Supplement: Figure S3 — Correlation between protein and gene expression levels of the SlETR1-SlETR7, SlCTR1-SlCTR3 and SlEIN2. Significant correlations are represented with an asterisk in the chart title and non-significant correlation with the letters N.S. The protein, gene expression data and their standard errors were normalized for visualization. [file Presentation_3.pptx]
