# Supplementary material for: Ethylene Receptors, CTRs and EIN2 Target Protein Identification and Quantification Through Parallel Reaction Monitoring During Tomato Fruit Ripening
Source: Front Plant Sci. 2018 Nov 8;9:1626. doi: 10.3389/fpls.2018.01626 (PMC6235968; doi:10.3389/fpls.2018.01626)
Supplement: Table S4 — Amino acid sequences of the proteins SlETR1-SlETR7, SlCTR1-SlCTR3 and SlEIN2 obtained from Uniprot (Bateman et al., 2015). Their Uniprotannotated transmembrane domains are underlined, their possible phosphorylation sites are highlighted in green and their GAF domain, kinase domain and response regulatory domains are represented in green, blue and orange fonts, respectively. The peptides used for the quantifications of the proteins in the current study are highlighted in yellow. [file Table_4.docx]

Supplementary Table 4: Amino acid sequences of the proteins SlETR1-SlETR7, SlCTR1-SlCTR3 and SlEIN2 obtained from Uniprot (Bateman et al., 2015). Their Uniprot annotated transmembrane domains are underlined, their possible phosphorylation sites are highlighted in green and their GAF domain, kinase domain and response regulatory domains are represented in green, blue and orange fonts, respectively. The peptides used for the quantifications of the proteins in the current study are highlighted in yellow.

**SlETR1**

>sp|Q41342|ETR1_SOLLC Ethylene receptor 1 OS=Solanum lycopersicum OX=4081 GN=ETR1 PE=1 SV=1

MGSLLRMNRLLSSIVESCNCIIDPQLPADDLLMKYQYISDFFIALAYFSIPVELIYFVKK

SAVFPYRWVLVQFGAFIVLCGATHLINLWTFNMHTRNVAIVMTTPKALTALVSCITALML

VHIIPDLLSVKTRELFLKKKAAQLDREMGIIRTQEETGRHVRMLTHEIRSTLDRHTILKT

TLVELGRTLALEECALWMPTRTGLELQLSYTLRHQNPVGLTVPIQLPVINQVFGTNHVVK

ISPNSPVARLRPAGKYMPGEVVAVRVPLLHLSNFQINDWPELSTKRYALMVLMLPSDSAR

QWHVHELELVEVVADQVAVALSHAAILEESMRARDLLMEQNVALDLARREAEMAVRARND

FLAVMNHEMRTPMHAIIALSSLLQETDLTPEQRLMVETILKSSNLLATLINDVLDLSRLE

DGSLQLDIGTFNLHALFREVHSLIKPIASVKKLFVTLSLSSDLPEYVIGDEKRLMQILLN

VVGNAVKFSKEGNVSISAFVAKSDSLRDPRAPEFFAVPSENHFYLRVQIKDTGIGITPQD

IPNLFSKFTQSQALATTNSGGTGLGLAICKRFVNLMEGHIWIESEGLGKGSTAIFIIKLG

IPGRANESKLPFVTKLPANHTQMSFQGLKVLVMDENGVSRMVTKGLLTHLGCDVTTVGSR

DECLRVVTHEHKVVIMDVSMQGIDCYEVAVVIHERFGKRHGRPLIVALTGNTDRVTKENC

MRVGMDGVILKPVSVYKMRSVLSELLEHGVVLES

**SlETR2**

>sp|O49187|ETR2_SOLLC Ethylene receptor 2 OS=Solanum lycopersicum OX=4081 GN=ETR2 PE=2 SV=1

MDCNCFDPLLPADELLMKYQYISDFFIAVAYFSIPIELVYFVQKSAVFPYRWVLVQFGAF

IVLCGATHLINLWTSTPHTRTVAMVMTTAKFSTAAVSCATAVMLVHIIPDLLSVKTRELF

LKNKAAELDREMGLIRTQEETGRYVRMLTHEIRSTLDRHTILKTTLVELGRALQLEECAL

WMPTRTGVELQLSYTLHHQNPVGFTVPIQLPVINQVFSANCAVKISPNSAVARLRPTRKY

IPGEVVAVRVPLLHLSNFQTNDWPELSPKSYALMVLMLPSNSARQWHVHELELVDVVADQ

VAVALSHAAILEESMRARDLLIEQNVALDLARREAETAVRARNDFLGVMNHEMRTPMHAV

VALSSLLQESELIPEQRLMVETILKSSNLLATLINDVLDLSRLEDGSLQLDVGTFNLHAL

FREVLNLIKPVAAVKKLFVTLSLSSDFPEVAIGDEKRLMQILLNVVGNAVKFSKEGSVSV

SAVNAKSESLIDPRAPEFFPVQSENHFYLRVQVKDTGSGINPQDFPKLFCKFAQNQEPAT

KNSAGTGLGLAICKRFVNLMEGHIWIESEGVGKGSTAIFIVKLGIPGRLNESKLPFTAGL

PANHMQMTFQGLKVLVMDDNGFSRMVTKSLLVHLGCDVTTIGSGDECLRILTREHKVLIM

DASITGMNCYDVAVSVHEKFGKRLERPLIVALTGNTDQVTKENCLRVGMDGVILKPVSID

KMRSVLSGLLEHGTVL

**SlETR3**

>tr|Q41341|Q41341_SOLLC Ethylene receptor neverripe OS=Solanum lycopersicum OX=4081 GN=Never-ripe PE=1 SV=1

MESCDCIEALLPTGDLLVKYQYLSDFFIAVAYFSIPLELIYFVHKSACFPYRWVLMQFGA

FIVLCGATHFISLWTFFMHSKTVAVVMTISKMLTAAVSCITALMLVHIIPDLLSVKTREL

FLKTRAEELDKEMGLIIRQEETGRHVRMLTHEIRSTLDRHTILKTTLVELGRTLDLAECA

LWMPCQGGLTLQLSHNLNNLIPLGSTVPINLPIINEIFSSPEAIQIPHTNPLARMRNTVG

RYIPPEVVAVR VPLLHLSNFTNDWAELSTRSYAVMVLVLPMNGLRKWREHELELVQVVAD

QVAVALSHAAILEDSMRAHDQLMEQNIALDVARQEAEMAIRARNDFLAVMNHEMRTPMHA

VIALCSLLLETDLTPEQRVMIETILKSSNLLATLINDVLDLSRLEDGILELENGTFNLHG

ILREAVNLIKPIASLKKLSITLALALDLPILAVGDAKRLIQTLLNVAGNAVKFTKEGHIS

IEASVAKPEYARDCHPPEMFPMPSDGQFYLRVQVRDTGCGISPQDIPLVFTKFAESRPTS

NRSTGGEGLGLAICRRFIQLMKGNIWIESEGPGKGTTVTFVVKLGICHHPNALPLLPMPP

RGRLNKGSDDLFRYRQFRGDDGGMSVNAQRYQRSL

**SlETR4**

>tr|Q9XET8|Q9XET8_SOLLC Ethylene receptor OS=Solanum lycopersicum OX=4081 GN=ETR4 PE=2 SV=1

MLRTLASALLVLSFFVSLSAADNGFPRCNCDDEGFWSIESILECQKISDLFIAIAYFSIP

IELLYFVSCSNFPFKWVLFQFIAFIVLCGMTHLLNFWTYYGQHPFQLMLALTIFKVLTAL

VSFATAITLITLFPMLLKVKVREFMLKKKTWDLGREVGLIKMQKEAGWHVRMLTQEIRKS

LDRHTILYTTLVELSKTLDLHNCAVWKPNENKTEMNLIHELRDSSFNSAYNLPIPR SDPD

VIQVKESDGVKILDADSPLAVASSGGSREPGAVAAIRMPMLKVSNFKGGTPELVPECYAI

LVLVLPSEQGRSWCSQEIEIVRVVADQVAVALSHAAILEESQHMRETLEEQNRALEQAKQ

DALRASQARNAFQMVMSHGLRRPMHSILGLLSLLQDEKLGNEQRLLVDSMVKTSNVVSTL

IDDVMDTSTKDNGRFPLEMRYFQLHSMIKEAACLAKCLCAYRGYNISIEVDKSLPNHVLG

DERRVFQVILHMVGNLLKDPNGGLLTFRVLPESVSREGIGGAWRTRRSNSSRDNAYIRFE

VGTSNNHSQPEGTMLPHYRPKRCSKEMDEGLSFTVCRKLVQLMQGDIWVIPNPEGFDQSM

AVVLGLQLRPSIAIGIPEYGESSDHSHPHSLLQGVKVLLADYDDVNRAVTSKLLEKLGCS

VSAVSSGRDCIGVLSPAVSSFQIVLLDLHLPDLDGFEVTMRIRKFGSHNWPLIVGLTATA

DENVTGRCLQIGMNGLIRKPVLLPGIADELQRVLLRGSRMM

**SlETR5**

>tr|Q9XET9|Q9XET9_SOLLC Ethylene receptor OS=Solanum lycopersicum OX=4081 GN=ETR5 PE=2 SV=1

MLAMLRLLFLVLLISLVIISVSANDGEFFNCCDEDGFWSIHTILDCQKVSDFFIAVAYFS

IPLELLYFISRSNLPFKWVLVQFIAFIVLCGLTHLLNGWTYNPHPSFQLILSLTVAKILT

ALVSCATAITLLTLIPLLLKIKVRELFLAQNVLELDQEVGMMKKQTEASMHVRMLTHEIR

KSLDKHTILYTTLVELSKTLKLQNCAVWMPNESRSQMNLTHELSPSSAAESHRSLSINDP

DVLEITKNKGVRILRQDSVLAASSSGGSGEPCAVAAIRMPLLRASDFKGGTPELVDTRYA

ILVLVLSSVDERVWSYDEMEIVEVVADQVAVALSHATVLEESQTMREKLEMRNRVLQQAQ

ENAMKASQARTSFQKVMNNGMRRPMHSILGLLSIFQDEKASSDQRMIVDTMVKTSTVLST

LINDAMEISAKDDGRFPVEMKPFQLHLLVREASCLVKCLCVYKGFGFSTDVPTSLPNQVM

GDEKRTFQVLLHMVGHLLNVSIGKGSVIFRVVLETGAETGNDKVWGTRRPSTTDEYVTIK

FEIEVSLEGSQSDSSISTIHFGGRRHNSKEVTEGLSFNMCKKLVQMMQGNIWMSSNAQGH

AQGMTLILRFQKQSSFRKRMFEYRNPLEQPISSTMFRGLHVLLTDDDDVNRLVTRKLLEK

LGCQVTAVSTGFQCLSALGPSLTTFQVLILDLQMPEMDGYEVALRVRKFRSRSWPLIIAL

TASSEEQVWEKCLQVGMNGLIRKPVLLQGLADELQRLLQRGGGGDGL

**SlETR6**

>tr|Q5QG21|Q5QG21_SOLLC Ethylene receptor OS=Solanum lycopersicum OX=4081 GN=ETR6 PE=3 SV=1

MMKKVVSWLLFLSIVASLWVVDGYIECPCDDSDAFFSMETMLFVQKAGDLGIAVAYFSIP

IEIIYFVSCSSFPFKWVLFQFGAFIVLCGLTHFLTFLTHFGKYTFHLILALIVCKLLTAL

VSMLTAITLMNLIPLLLKAKAREFMLRRKNRELDREVEKIKQLEELGLHVRMLTNEIRKS

IDRHTILYTTLVGLSKLLSLQNCVIWMPNENRTEMKLTHDTTRENVSSVYNVPIPISDRE

VKEIKGSDDVKILGADSRLAAASSRGSCEPESVAAIRIPMLTVSNFRGETREIVSQCYAI

LVLVQPCGHGRFWLNQEVEIVRAAADQVAVALSHAAVVEESEYIKDRLMEQNQALQKARE

EALRASQARSSFQTVMSHRLRRPMHSILGLLSMLQEQKLRDEQQLLVHSIIKSSNVVSTL

MDDVIVTSTKENVKFPLEMKHFQLHSLIREAACTAKSLCMYKGYNITIEVEKSFPNKVMG

DERRFFQVLLHIIGNLLNGIHGGHLTFKVLSASENDVSWKTPRSNSSNDIVYIKFEICTK

FNRSQSEITPAPPTYDTEEIEESLSFAVCRKLVHLMQGDIFIIRNLADFDQGMAVIVGFQ

RQPLIPLGMSEYVESSNPTYPHPVLRGVEVLLADYDDSNRAVTKKMLEKLGCIVTLVSSG

YECLGAVGPVVSSLQIILLDLHLPDLDGFEVTMRLRKHRRQTWPLIIGLAAITDEDIRKC

LKIGMNGIICKPLLLSGLADELQKVLLHANRGMP

**SlETR7**

>tr|K4C2I4|K4C2I4_SOLLC Ethylene receptor OS=Solanum lycopersicum OX=4081 PE=3 SV=1

MATDSEFSNCNCDEEGVFWNIHTILDCQKVSDFLIAIAYFSIPLELLYFISCSDVPFKWV

LVQFIAFIVLCGLTHLLNGLTYSAHPSFQLIMSLTVAKILTALVSCATAITLLTLFPMLL

KVKVRELFLTQNVLELDQEVGMMKKQKEVYTHVRMLTREIRKSLDKHTILYTTLVELSKT

LNLQNCAVWMPNEDRSLMNLTHGLSPGSAVEYHRSLPIDDPDVLEITKNKGVRILRQDSV

LAAASSGGPGEPCTVAAIRMPLLCASDFKGGTPELVDTRYAILVLVIPGANDDCSHNEME

IVEVVADQVAVALSHATVLEESQLMREKLEARNGLLQQAKENAVKASQARNSFQKVMNNG

MRRPMHSVLGLLSILQDENTSSNQKIIIDTMVRTSTVLSNLINDAMDIPDKDEGRFPVEM

MPFQLHSLIREASCLVKCLCVYKGFRFSTDVPNSLPNLVMGDEKRTFQVILHMVGHLLNI

SSGRGSVVFKVILESGIEGGNDKLQGARKHSVFDEYVTIKFEIEVSRGGSQTDSSISTSH

FGGKRYNSKELKEGMSFSMCKKLVQMMQGNVWMPSNTDGHAQKMTLILRFLKQSSFRKHM

FELVHPLEQAISSSTFKGLQVLLADDDDVNRMVTKKLLQKLGCQVIAVSSGFQCLSAMGH

STTSIQVVILDLHMPEMDGFEVTTRVRKFHSRSWPLIIALSSTSEQQVWDRCLQVGINGL

IRKPVLLQGMAEELQRVLQRAGEGF

**SlCTR1**

>tr|O24027|O24027_SOLLC Ethylene-responsive protein kinase Le-CTR1 OS=Solanum lycopersicum OX=4081 GN=ER50 PE=2 SV=1

MSGRRSSYTLLNQIPNDNFFQPPAPKFSAGAGVVPYGESSSAEKNRGKVFDLDLMDQRMM

QSHNRVGSFRVPGSIGSQRQSSEGSFGGSSLSGENYVGTSFGHKNEGCGSSVARSWAQQT

EESYQLQLALAIRLSSEATCADSPNFLDPVTDVLASRDSDSTASAVTMSHRLWINGCMSY

FDKVPDGFYWIYGMDPYVWALCSVVQESGRIPSIESLRAVDPSKAPSVEVILIDRCNDLS

LKELQNRIHSISPSCITTKEAVDQLAKLVCDHMGGAAPAGEEELVSMSKGCSNDLKDRFG

TIVLPIGSLSVGLCRHRALLFKVLADIIDLPCRIAKGCKYCNSSDASSCLVRFEHDREYL

VDLIGKPGVLSEPDSLLNGPSSISIPSPLRFPRYRQVEPTTDFRSLAKQYFLDSQSLNLL

FDDSSAGAAADGDAGQSDRSCIDRNNVVSSSSNRDEISQLPLPPLNAWKKGRDKESQLSK

MYNPRSMLNPVNMDEDQVLVKHVPPFREDAQSPMTRPDTVNDTRFLAGGGHVVSAIPSEE

LDLDVEEFNIPWNDLILMEKIGAGSFGTVHRGDWHGSDVAVKILMEQDFHAERLKEFLRE

VAIMKRLRHPNIVLFMGAVIQPPNLSIVTEYLSRGSLYRLLHKPGAREVLDERRRLCMAY

DVANGMNYLHKRNPPIVHRDLKSPNLLVDKKYTVKICDFGLSRFKANTFLSSKTAAGTPE

WMAPEVIRDEPSNEKSDVYSFGVILWELATLQQPWNKLNPPQVIAAVGFNRKRLDIPSDL

NPQVAIIIEACWANEPWKRPSFSTIMDMLRPHLKSPLPPPGHTDMQLLS

**SlCTR2**

>tr|O65833|O65833_SOLLC TCTR2 protein OS=Solanum lycopersicum OX=4081 GN=TCTR2 PE=2 SV=2

MKHIFKKLHHSNRSNDAQSTSSISSSSSPASSLSSASCTTDHRNSNSVSQSPLSPSTIST

ASTTTTPAAPVGAGGGGGGGNLSTINRQQDYYTSEEEYQVQLALALSVSSSQSQDPFPSD

VNSSNGHGVGRTAVDLARDREDAAADLLSRQYWDYGVMDYEEKVVDGFYDVYNLFTDPAS

RGKMPSLSELETNPGTSNFEGVIINQRIDPSLEELMQIAHCITLDCPASEISLLVLRLSE

LVTGHLGGPVKDANIILAKWMEISTELRTSLHTSVLPIGSLKIGLSRHRALLFKVLADHV

GIPCRLVKGSHYTGVEDDAVNIVKLPNDSEFLVDLMGAPGTLIPADVLSAKDASFNSPKL

NKIPSLPSNSHSGVSYPRRNLLSGQNSVLGDDFSGRSKPEKIESVHSISDAGGSSTAGSS

GINKRPSSNQVDWTSPLAIGTSLYKGGRGPNAAGDGLRLNVNVVPYDQNNPEDPKNLFAD

LNPFQIKGSGNTLLQKNPARNKVSELQQPINTLIPGRPPAPMMWKNRYAPNEVPRKNESD

SEGLFPKKNGGSSGYNISSIASTSSNIPQKSSTDTSRLHGNSRPAYRGNDEVASTRNNSS

ILSAELEFRRLSVQNSQNNNRETSQWEGHSLQSDDLNRTQAYGDDIIVESDHTRNLQAQS

IGTNIKLKEPENPTSSGNLGPSQVDPVFDDVGDCEIPWEDLVIGERIGLGSYGEVYHADW

NGTEVAVKKFLDQDFSGAALAEFKREVRIMRRLRHPNVVRFMGAITRPPHLSIITEFLPR

GSLYRIIHRPHFQIDERQKIKMALDVAKGMDCLHTSNPTIVHRDLKSPNLLVDTDWNVKV

CDFGLSRLKHNTFLSSKSTAGTPEWMAPEVLRNEPSNEKCDIYSFGVILWELATLRLPWS

GMNPMQVVGAVGFQNKRLEIPKELDPIVARIIWECWQTDPNLRPSFAQLTVALTPLQRLV

IPAYVDQLNSRLPQEISVNSTP

**SlCTR3**

>tr|Q5YKK5|Q5YKK5_SOLLC CTR1-like protein kinase OS=Solanum lycopersicum OX=4081 GN=CTR3 PE=2 SV=1

MEMSTRRSNYTLLSQVADDNYLPPPPKYSVTGGGGGGGGVAPYYESHSGEKGKGKTGDNR

GFDWDLSDHRSNMMQASNRIGAAAFPGSIGLQRQSSGSSFGESSISGEYYMPSLSNAEAS

FGYLNDGGGGAEVRMKPLEANLFGGSSSKSWAQQTEESYQLQLALALRLSSEATCADDPN

FLDHVPDESASRASASAASAETLSHRFWVNGCLSYFDKVPDGFYLIHGMDPYVWIVCSDL

QENARVPSIESMRAVDPSVVPSVEVILIDRRTDPSLKELQNRIHSLSPTCGTTKEVVDQL

AQLVCSHMGGATSAGEDELVPLWKECSYELKDCLGSTVLPIGSLSVGLCRHRALLFKVLA

DAIGLPCRIAKGCKYCNRADASSCLVRFGPDREYLVDLIGSPGCLCEPDSSLNGPSSISI

SSPLRFPRFREVEPTTDFRSLAKQYFSDCQSLNLVFEESSAGAAVDGDAGQTDRNNIERN

SAVTGPSNRDEVSRLPVPAIRDMAPVKYVRPVLHGDTQLSDPRDIGNDMRFLERGSQLVP

SKISRDIALEIEDFDIPWEDLVLKERIGAGSFGTVHRADWNGSDVAVKILMEQDFHAERF

KEFLREVAIMKRLRHPNIVLFMGAVTQRPNLSIVTEYLSRGSLYRLLHKPGAREVLDERR

RLSMAYDVAKGMNYLHKRNPPIVHRDLKSPNLLVDKKYTVKVCDFGLSRLKANTFLSSKS

AAGTPEWMAPEVLRDEPSNEKSDVYSFGVILWELATLQQPWSNLNPAQVVAAVGFKGKRL

DIPRDLTPQVASIIEACWAKEPWKRPSFAAIMDMLRPLIKPPVTPPQPGRTDTQLIA

**SlEIN2**

>tr|Q6Q2C1|Q6Q2C1_SOLLC Ethylene signaling protein OS=Solanum lycopersicum OX=4081 GN=EIN2 PE=2 SV=2

MESETLTREYRRPSMLQRVLSASVPMLLIAVGYVDPGKWAAMVDGGARFGFDLVMLVLLF

NFAAILCQYLSACIALVTDRDLAQICSEEYDKVTCIFLGIQAEVSMIALDLTMVLGTAHG

LNVVFGVDLFSCVFLTATGAILFPLLASLLDNGSAKFLCIGWASSVLLSYVFGVVITLPE

TPFSIGGVLNKFSGESAFALMSPLGASIMPHNFYLHSSIVQQGKESTELSRGALCQDHFF

AIVFIFSGIFLVNYAAMNSAANVSYSTGLLLLTFQDTLSLLDQVFRSSVAPFTIMLVTFI

SNQVTPLTWDLGRQAVVHDLFGMDIPGWLHHVTIRVISIVPALYCVWSSGAEGLYQLLIL

TQVVVALVLPSSVIPLFRVASSRSIMGIHKISQLMEFLSLGTFIGLLGLKIIFVIEMIFG

NSDWVNNLKWNIGSSVSTPYFFLLIAASLCLCLMLWLAVTPLKSASSRFDAQAFLQTHVP

EPYSECNQLGASNAMFGLVEGSSQKQEGAFHVEKSLVSHPDLSTKDPDQLLPESLLDFEK

VHQLATIDESKSETTFSAPAVVHPEVPVSAGASPSVKSVCNEVSGVVSVDTSVFNTETVD

VAEKTLRIEGDMANDRDDGDSWEEPEEAIKGVSENAQSFISDGPGSYKSLSGKLEDTGSG

TGSLSRLAGLGRAARRQLTEALNEFWGQLFDYHGVATAEAKSKKLDIILGLDSKMNPKPA

PASLKVESSAYIPSGSARIPEPLINSHVYSPKQQFASNIVDSAYRVPKEPSSTSSMWSNH

MKLVGAYVQSSNSNMLDSGERRYSSMRIPATSAGYDQQPATVHGYQITAYLNQLAKERGS

DYLNGQLESPSPRSVSSLTSNYAEPLARVSGQKPQSGVSSRAPPGFGNVPVGRNNSMQPT

NTTSVDHSSTETAESVAGSANSKKYYSLPDISGRYVPRQDSIVSDARAQWYNSMGFGQSG

GRSTYEQAYMSGSLRAGGPQRYEHSPKVCRDAFSLQYSSNSGTGSLWSRQPFEQFGVAGK

PDVGSGDHGTVLSSSAQESTSTVDLEAKLLQSFRSCIVKLLKLEGSEWLFRQDDGADEDL

IGRIAAREKFLYEAETREISRLTNIGESHFSSNRKPGSAPKPEEMDYTKFLVMSVPHCGE

GCVWKVDLIISFGVWCIHRILELSLMESRPELWGKYTYVLNRLQGIVDLAFSKPHSPTSH

CFCLQIPAGRQQKASPPPISNGNLPPQAKQGRGKCTTAAMLLEMIKDVETAISCRKGRTG

TAAGDVAFPKGKENLASVLKRYKRRLSNKPVGNQEVAGVAGPRKVTLSASSPPFVL

**References**

Bateman, A., Martin, M. J., O’Donovan, C., Magrane, M., Apweiler, R., Alpi, E., et al. (2015). UniProt: a hub for protein information. Nucleic Acids Res. 43, D204–D212. doi:10.1093/nar/gku989.
