# Supplementary material for: Ethylene Receptors, CTRs and EIN2 Target Protein Identification and Quantification Through Parallel Reaction Monitoring During Tomato Fruit Ripening
Source: Front Plant Sci. 2018 Nov 8;9:1626. doi: 10.3389/fpls.2018.01626 (PMC6235968; doi:10.3389/fpls.2018.01626)
Supplement: Table S5 — Cluster alignment of Q9S814 (AtEIN2) and Q6Q2C1 (SlEIN2) with the alignment tool of Uniprot. The C-terminal part of AtEIN2 is highlighted in green based on the results of Qiao et al. (2012). The two peptides of SlEIN2 identified and quantified in this work are highlighted in yellow. [file Table_5.docx]

Supplementary Table 5: Cluster alignment of Q9S814 (AtEIN2) and Q6Q2C1 (SlEIN2) with the alignment tool of Uniprot. The C-terminal part of AtEIN2 is highlighted in green based on the results of Qiao et al. (2012). The two peptides of SlEIN2 identified and quantified in this work are highlighted in yellow.

CLUSTAL O(1.2.4) multiple sequence alignment

SP|Q9S814|EIN2_ARATH MEAEIV-NVRPQLGFIQRMVPALLPVLLVSVGYIDPGKWVANIEGGARFGYDLVAITLLF 59

TR|Q6Q2C1|Q6Q2C1_SOLLC MESETLTREYRRPSMLQRVLSASVPMLLIAVGYVDPGKWAAMVDGGARFGFDLVMLVLLF 60

SP|Q9S814|EIN2_ARATH NFAAILCQYVAARISVVTGKHLAQICNEEYDKWTCMFLGIQAEFSAILLDLTMVVGVAHA 119

TR|Q6Q2C1|Q6Q2C1_SOLLC NFAAILCQYLSACIALVTDRDLAQICSEEYDKVTCIFLGIQAEVSMIALDLTMVLGTAHG 120

SP|Q9S814|EIN2_ARATH LNLLFGVELSTGVFLAAMDAFLFPVFASFLENGMANTVSIYSAGLVLLLYVSGVLLSQSE 179

TR|Q6Q2C1|Q6Q2C1_SOLLC LNVVFGVDLFSCVFLTATGAILFPLLASLLDNGSAKFLCIGWASSVLLSYVFGVVITLPE 180

SP|Q9S814|EIN2_ARATH IPLSMNGVLTRLNGESAFALMGLLGASIVPHNFYIHSYFAGESTSSSDVDKSSLCQDHLF 239

TR|Q6Q2C1|Q6Q2C1_SOLLC TPFSIGGVLNKFSGESAFALMSPLGASIMPHNFYLHSSIVQQGKESTELSRGALCQDHFF 240

SP|Q9S814|EIN2_ARATH AIFGVFSGLSLVNYVLMNAAANVFHSTGLVVLTFHDALSLMEQVFMSPLIPVVFLMLLFF 299

TR|Q6Q2C1|Q6Q2C1_SOLLC AIVFIFSGIFLVNYAAMNSAANVSYSTGLLLLTFQDTLSLLDQVFRSSVAPFTIMLVTFI 300

SP|Q9S814|EIN2_ARATH SSQITALAWAFGGEVVLHDFLKIEIPAWLHRATIRILAVAPALYCVWTSGADGIYQLLIF 359

TR|Q6Q2C1|Q6Q2C1_SOLLC SNQVTPLTWDLGRQAVVHDLFGMDIPGWLHHVTIRVISIVPALYCVWSSGAEGLYQLLIL 360

SP|Q9S814|EIN2_ARATH TQVLVAMMLPCSVIPLFRIASSRQIMGVHKIPQVGEFLALTTFLGFLGLNVVFVVEMVFG 419

TR|Q6Q2C1|Q6Q2C1_SOLLC TQVVVALVLPSSVIPLFRVASSRSIMGIHKISQLMEFLSLGTFIGLLGLKIIFVIEMIFG 420

SP|Q9S814|EIN2_ARATH SSDWAGGLRWNTVMGTSIQYTTLLVSSCASLCLILWLAATPLKSASNRAEAQIWNMDAQN 479

TR|Q6Q2C1|Q6Q2C1_SOLLC NSDWVNNLKWNIGSSVSTPYFFLLIAASLCLCLMLWLAVTPLKSASSRFDAQAFLQTHVP 480

SP|Q9S814|EIN2_ARATH ALSYPSVQEEE-----IERTETRRNEDESIVRLESRVKDQLDTTSVTSSVYDLPENILMT 534

TR|Q6Q2C1|Q6Q2C1_SOLLC E-PYSECNQLGASNAMFGLVEGSSQKQEGAFHVEKSLVSHPDLS-TKDPDQLLPESLLDF 538

SP|Q9S814|EIN2_ARATH DQEIRSSPPEERELDVKYSTSQVSSLKEDSDVKE-QSVLQSTVVNEVSDKDLIVETKMAK 593

TR|Q6Q2C1|Q6Q2C1_SOLLC EKVHQLATIDESKSETTFSAPAVVHP--EVPVSAGASPSVKSVCNEVSGV-VSVDTSVFN 595

SP|Q9S814|EIN2_ARATH IEPMSPVEKIVSMENNSKFIEKDVEGVSWETEEA-TKAAPTSNFTVGSDGPPSFRSLSG- 651

TR|Q6Q2C1|Q6Q2C1_SOLLC TETVDVAEKTLRI--EGDMANDRDDGDSWEEPEEAIKGVSENAQSFISDGPGSYKSLSGK 653

SP|Q9S814|EIN2_ARATH --EGGSGTGSLSRLQGLGRAARRHLSAILDEFWGHLYDFHGQLVAEARAKKLDQLFGTDQ 709

TR|Q6Q2C1|Q6Q2C1_SOLLC LEDTGSGTGSLSRLAGLGRAARRQLTEALNEFWGQLFDYHGVATAEAKSKKLDIILGLDS 713

SP|Q9S814|EIN2_ARATH KSASSMKADSFGKDISSGYCMSPTAKGMDSQMTSSLYDSLKQQRTPGSIDSLYGLQRGS- 768

TR|Q6Q2C1|Q6Q2C1_SOLLC KMNPKPAPASLKVE-SSAYIPSGSARIPEPLINSHVY-SPKQQFASNIVDSAYRVPKEPS 771

SP|Q9S814|EIN2_ARATH -SPSPLVNRMQMLGAYGNTTNNNNAYELSERRYSSLRAPSSSEGWEHQQPATVHGYQMKS 827

TR|Q6Q2C1|Q6Q2C1_SOLLC STSSMWSNHMKLVGAYVQSS-NSNMLDSGERRYSSMRIPATSAGYD-QQPATVHGYQITA 829

SP|Q9S814|EIN2_ARATH YVDNLAKERLEA-LQSRGEIPTSRSMALGTLSYTQQLALALKQKSQNGLTPGPAPGFENF 886

TR|Q6Q2C1|Q6Q2C1_SOLLC YLNQLAKERGSDYLNGQLESPSPRSVSSLTSNYAEPLARVSGQKPQSGVSSRAPPGFGNV 889

SP|Q9S814|EIN2_ARATH AGSRSISRQSERSYYGVPSSGNTDTVGAAVANEKKYSSMPDISGLSMSARNMHLPNNKSG 946

TR|Q6Q2C1|Q6Q2C1_SOLLC PVGRNNSMQPTNTTSVDHSSTETAESVAGSANSKKYYSLPDISGRYVPRQDSIVSDARAQ 949

SP|Q9S814|EIN2_ARATH YWDPSSGGGGYGASYGRLSNESSLYSNLGSRVGVPSTYDDISQSRGGYRDAYSLPQSATT 1006

TR|Q6Q2C1|Q6Q2C1_SOLLC WY-NSMG---FGQSGGRSTYEQ-AYMSGSLRAGGPQRYEH---SPKVCRDAFSLQYSSNS 1001

SP|Q9S814|EIN2_ARATH GTGSLWSRQPFEQFGVAERNGAVGEELRNRSNPINIDNNASSNVDAEAKLLQSFRHCILK 1066

TR|Q6Q2C1|Q6Q2C1_SOLLC GTGSLWSRQPFEQFGVAGKPDVGSGDHGTV--LSSSAQESTSTVDLEAKLLQSFRSCIVK 1059

SP|Q9S814|EIN2_ARATH LIKLEGSEWLFGQSDGVDEELIDRVAAREKFIYEAEAREINQVGHMGEP----------- 1115

TR|Q6Q2C1|Q6Q2C1_SOLLC LLKLEGSEWLFRQDDGADEDLIGRIAAREKFLYEAETREISRLTNIGESHFSSNRKPGSA 1119

SP|Q9S814|EIN2_ARATH -----------LISSVPNCGDGCVWRADLIVSFGVWCIHRVLDLSLMESRPELWGKYTYV 1164

TR|Q6Q2C1|Q6Q2C1_SOLLC PKPEEMDYTKFLVMSVPHCGEGCVWKVDLIISFGVWCIHRILELSLMESRPELWGKYTYV 1179

SP|Q9S814|EIN2_ARATH LNRLQGVIDPAFSKLRTPMTPCFCLQIPASHQR--ASPTSANGMLPPAAKPAKGKCTTAV 1222

TR|Q6Q2C1|Q6Q2C1_SOLLC LNRLQGIVDLAFSKPHSPTSHCFCLQIPAGRQQKASPPPISNGNLPPQAKQGRGKCTTAA 1239

SP|Q9S814|EIN2_ARATH TLLDLIKDVEMAISCRKGRTGTAAGDVAFPKGKENLASVLKRYKRRLSNKPVGMNQDGPG 1282

TR|Q6Q2C1|Q6Q2C1_SOLLC MLLEMIKDVETAISCRKGRTGTAAGDVAFPKGKENLASVLKRYKRRLSNKPVGNQEVAGV 1299

SP|Q9S814|EIN2_ARATH S-RKNVTAYGSLG---- 1294

TR|Q6Q2C1|Q6Q2C1_SOLLC AGPRKVTLSASSPPFVL 1316

**References**

Qiao, H., Shen, Z., Huang, S. -s. C., Schmitz, R. J., Urich, M. A., Briggs, S. P., et al. (2012). Processing and Subcellular Trafficking of ER-Tethered EIN2 Control Response to Ethylene Gas. *Science (80-. ).* 338, 390–393. doi:10.1126/science.1225974.
